# Sonic Hedgehog signaling limits atopic dermatitis via Gli2-driven immune regulation

**DOI:** 10.1172/JCI125170

**Published:** 2019-07-02

**Authors:** Eleftheria Papaioannou, Diana C. Yánez, Susan Ross, Ching-In Lau, Anisha Solanki, Mira Manilal Chawda, Alex Virasami, Ismael Ranz, Masahiro Ono, Ryan F. L. O’Shaughnessy, Tessa Crompton

**Affiliations:** 1 Great Ormond Street Institute of Child Health, University College London, London, United Kingdom.; 2 School of Medicine, Universidad San Francisco de Quito, Quito, Ecuador.; 3 Department of Respiratory Medicine and Allergy, King’s College London, London, United Kingdom.; 4 Department of Life Sciences, Imperial College London, London, United Kingdom.; 5 Blizard Institute, Queen Mary University of London, London, United Kingdom.

**Keywords:** Immunology, Inflammation, Adaptive immunity, Skin, T cells

## Abstract

Hedgehog (Hh) proteins regulate development and tissue homeostasis, but their role in atopic dermatitis (AD) remains unknown. We found that on induction of mouse AD, Sonic Hedgehog (Shh) expression in skin and Hh pathway action in skin T cells were increased. Shh signaling reduced AD pathology and the levels of Shh expression determined disease severity. Hh-mediated transcription in skin T cells in AD-induced mice increased Treg populations and their suppressive function through increased active transforming growth factor–β (TGF-β) in Treg signaling to skin T effector populations to reduce disease progression and pathology. RNA sequencing of skin CD4^+^ T cells from AD-induced mice demonstrated that Hh signaling increased expression of immunoregulatory genes and reduced expression of inflammatory and chemokine genes. Addition of recombinant Shh to cultures of naive human CD4^+^ T cells in iTreg culture conditions increased FOXP3 expression. Our findings establish an important role for Shh upregulation in preventing AD, by increased Gli-driven, Treg cell–mediated immune suppression, paving the way for a potential new therapeutic strategy.

## Introduction

Skin is in direct contact with the environment and acts as protective barrier from mechanical injuries and infections. Disruption of skin homeostasis and barrier function together with immune dysregulation result in inflammatory diseases of the skin, such as atopic dermatitis (AD), a chronic inflammatory disease with complex etiology characterized by dry scaly skin lesions and pruritus (1). The mechanisms that cause AD remain controversial, but Filaggrin, a terminal differentiation marker of keratinocytes, is important for skin barrier formation, and its deficiency promotes immune alterations which contribute to AD (2). Skin inflammation in AD changes through time, with expanded T helper 2 (Th2) and Th17 responses at early stages, and a partial switch to a Th1 response during chronic stages (1–3). CD4^+^CD25^+^Foxp3^+^ Tregs are abundant in skin, and in inflammation they infiltrate the dermis but lose their immunoregulatory function, further enhancing the inflammatory response (1, 4–8). The reasons why the CD4^+^CD25^+^Foxp3^+^ population in skin fails to suppress the immune response in AD is unclear, so identification of skin factors that promote immune regulation is important to preventing AD.

Here we investigate Hh signaling in chronic AD using a mouse model of hapten-induced dermatitis, with all characteristics of the disease. The Hh proteins Sonic (Shh), Dessert (Dhh), and Indian (Ihh) are morphogens that share a common signaling pathway and regulate development and tissue homeostasis (9–11). Binding to their receptor Patched1 (Ptch1) initiates the pathway by relieving Smoothened (Smo) inhibition. At the end of the pathway are the Gli transcription factors (Gli1, Gli2, and Gli3), which bind DNA at consensus Gli-binding sites. Gli1 acts only as a transcriptional activator, whereas Gli2 and Gli3 function as activators of transcription in the presence of Hh signaling or repressors in its absence, with some redundant or partially overlapping functions.

Shh is expressed in hair follicles and dysregulated Hh signaling in skin leads to basal cell carcinoma (BCC) (12, 13). Hh signaling regulates T cell development and T cell receptor (TCR) repertoire selection (14–21). Gli1 and Gli2 are expressed in peripheral T cells and Hh signaling can impair T cell activation (15, 17, 18, 22–25).

Here we use constitutive and conditional mutant mice to investigate epithelium-lymphocyte cross-talk in AD. We show that Shh upregulation and increased Hh signaling to T cells in skin play a critical role in preventing skin inflammation on induction of chronic AD. Thus, our study dissects a previously unknown signaling mechanism, mediated by the skin-derived morphogen Shh, which is required for and promotes Treg-dependent skin homeostasis.

## Results

### Induction of AD upregulates Shh in skin and increases Hh pathway activation in skin T cells.

**Figure 1:**
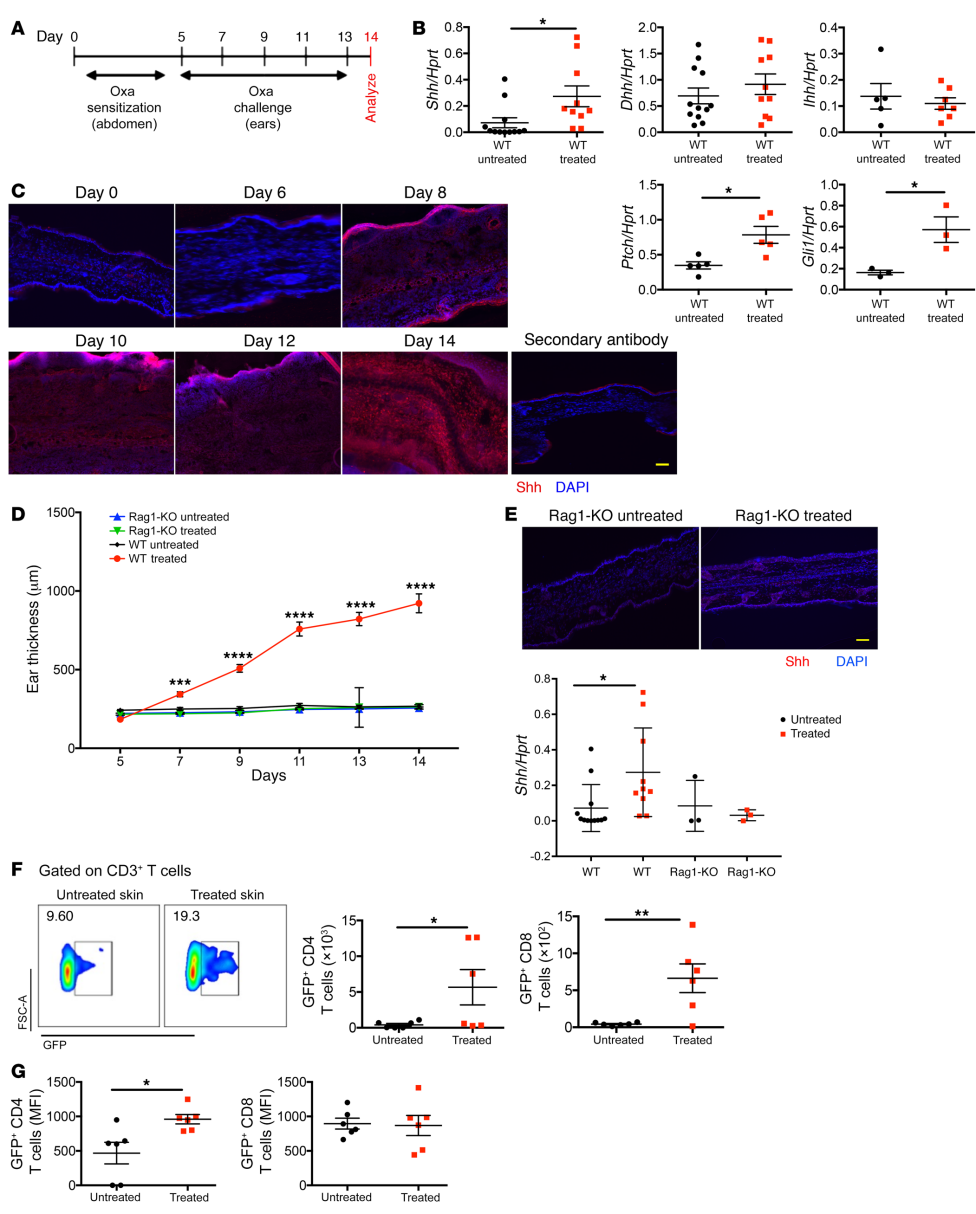
Shh upregulation and in vivo activation of the Hh signaling pathway upon induction of AD. Black circles, control mice; red squares, Oxa-treated mice. Each symbol represents an individual animal. (**A**) Sensitization and challenge scheme of Oxa treatment. (**B**) mRNA expression by QRT-PCR of Hh signaling components in whole ear skin homogenates from untreated WT and Oxa-treated WT mice. Data from 2 independent experiments. (**C**) Representative immunofluorescence staining images of Shh (red) expression in frozen skin sections from untreated WT (day 0) and Oxa-treated WT mice on days 6, 8, 10, 12, and 14 after initiation of the Oxa protocol. DAPI-stained nuclei are shown in blue. Scale bar: 100 μm (*n* = 5 mice per group). (**D**) Time course of ear thickness from Rag1-KO control (blue), Oxa-treated Rag1-KO (green), WT control (black), and Oxa-treated WT (red) mice (*n* = 6 per group). (**E**) Representative immunofluorescence staining images of Shh (red) expression in frozen skin sections from untreated Rag1-KO and Oxa-treated Rag1-KO mice on termination (day 14). DAPI-stained nuclei are in blue. Scale bar: 100 μm (*n* = 3 mice per group). Plot shows comparison of Shh mRNA expression in whole ear homogenates from control untreated Rag1-KO (*n* = 3) and Oxa-treated Rag1-KO (*n* = 3) with equivalent Shh expression data from WT from **B**. Data from 2 independent experiments. (**F**) Representative density plots of GFP expression in skin CD3^+^ T cells from untreated and Oxa-treated GBS-GFP transgenic mice, giving percentage of cells in GFP^+^ region shown. Plots show number of skin GFP^+^CD4^+^ and GFP^+^CD8^+^ T cells isolated from ears from untreated and Oxa-treated GBS-GFP transgenic mice. (**G**) MFI of GFP in skin GFP^+^CD4^+^ and GFP^+^CD8^+^ T cells from untreated and Oxa-treated GBS-GFP transgenic mice. In **B**, **E**–**G**, 2-tailed unpaired Student’s *t* test was used; in **D**, ANOVA was used. Plots show mean ± SEM. *P < 0.05, **P < 0.01, ***P < 0.001, ****P < 0.0001.

We used an oxazolone-induced (Oxa-induced) model of extrinsic AD in mice (Figure 1A), which generates T cell–driven skin inflammation in the ears, with all the features of chronic human AD, including swelling, T cell infiltration, and IgE and cytokine production (Supplemental Figure 1, A–E) (26). We examined expression of Hh pathway components in inflamed skin from Oxa-treated WT mice and in skin from control WT mice. *Shh* mRNA was significantly elevated in Oxa-treated compared with untreated skin, whereas there was no significant change in *Dhh* and *Ihh* transcripts (Figure 1B). *Ptch1* and *Gli1*, which are both Hh pathway components and target genes (9), were also upregulated in Oxa-treated skin compared with control (Figure 1B). Immunofluorescence staining of ear skin revealed increasing expression of Shh in dermis and epidermis of mice with AD, from day 8 of the protocol (Figure 1C). In contrast to Oxa treatment of WT mice, Oxa treatment of lymphocyte-deficient Rag1-KO mice failed to induce ear swelling (Figure 1D), downregulation of *Filaggrin*, or upregulation of *Ifng* and *Il4*, confirming the lymphocyte dependence of disease induction (Supplemental Figure 1F). Shh was not upregulated in the skin of the Rag1-KO mice, indicating that the increased expression of Shh on hapten treatment is dependent on lymphocyte-driven inflammation (Figure 1E).

To test if the Hh signaling pathway is active in skin T cells after AD induction, we used Gli binding site (GBS)–GFP reporter transgenic mice (27) to measure the proportion of T cells that show active Gli-mediated transcription. The percentage of CD3^+^ T cells that expressed GFP was higher in Oxa-treated skin compared with control (Figure 1F). There were significantly more GFP^+^CD8^+^ and GFP^+^CD4^+^ T cells, with elevated MFI of GFP in CD4^+^ T cells in Oxa-treated skin (Figure 1, F–G), indicating that not only did more skin CD4^+^ T cells undergo active Hh signaling after disease induction, but also that the level of Hh pathway activation was higher in individual CD4^+^ T cells. There was therefore an increase in both Shh expression in skin and in Gli-mediated transcription in skin T cells from Oxa-treated mice, suggesting that Hh signaling might be involved in AD.

### Shh mutation increases severity of AD whereas Gli3 mutation ameliorates chronic AD.

**Figure 2:**
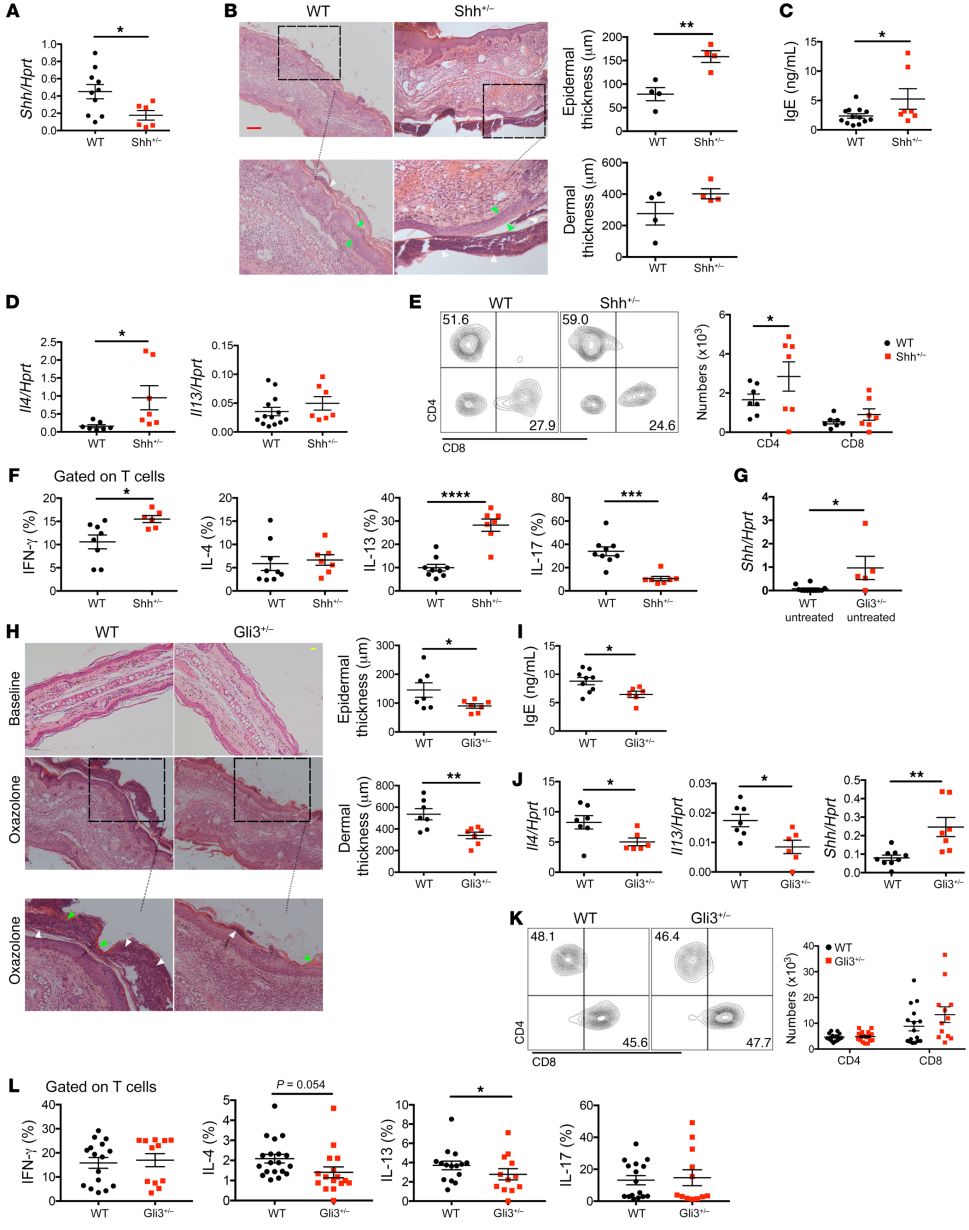
Shh mutation aggravates but Gli3 mutation ameliorates chronic AD. (**A**–**F**) Oxa-treated Shh^+/–^ mice (red) and WT littermates (black). (**A**) *Shh* expression (QRT-PCR) in ear homogenates from Oxa-treated WT and Shh^+/–^ mice. (**B**) Representative H&E images of ear sections from Oxa-treated WT (*n* = 4) and Shh^+/–^ (*n* = 4) mice (day 14), showing areas of hyperkeratosis (green arrows) and parakeratosis (white arrows). Scale bar: 100 μm. Plots show dermal and epidermal thickness. (**C**) Serum IgE concentration (ELISA) from Oxa-treated WT and Shh^+/–^ mice. (**D**) *Il4* and *Il13* expression (QRT-PCR) in whole ear homogenates from Oxa-treated WT and Shh^+/–^ mice. (**E**) Contour plot shows CD4 and CD8 expression, gated on CD45^+^CD3^+^γδTCR^–^ cells from ears of Oxa-treated WT and Shh^+/–^ mice. Plots show number of CD4^+^ and CD8^+^ T cells isolated from ears. (**F**) Percentage of skin T cells from Oxa-treated WT and Shh^+/–^ mice that express IFN-γ, IL-4, IL-13, and IL-17, measured by flow cytometry. (**G**) *Shh* expression (QRT-PCR) in whole ear homogenates from untreated WT (black) and Gli3^+/–^ (red) mice. (**H**–**L**) Data from 2 independent experiments using Oxa-treated WT (black) and Gli3^+/–^ (red) littermates. (**H**) Representative H&E images of ear sections from untreated (baseline) and Oxa-treated WT and Gli3^+/–^ mice (day 14), showing areas of hyperkeratosis (green arrows) and parakeratosis (white arrows). Scale bar: 100 μm. Plots show dermal and epidermal thickness. (**I**) Serum IgE concentration (ELISA) from Oxa-treated WT and Gli3^+/–^ mice. (**J**) *Il4*, *Il13*, and *Shh* expression (QRT-PCR) in whole ear homogenates from Oxa-treated WT and Gli3^+/–^ mice. (**K**) Contour plot shows CD4 and CD8 expression, gated on CD45^+^CD3^+^γδTCR^–^ cells isolated from Oxa-treated ears from WT and Gli3^+/–^ mice. Plots show number of CD4^+^ and CD8^+^ T cells isolated from ears. (**L**) Percentage of skin T cells from Oxa-treated WT and Gli3^+/–^ mice that express IFN-γ, IL-4, IL-13, and IL-17, measured by flow cytometry. Plots show mean ± SEM; each symbol represents an individual animal; 2-tailed unpaired Student’s *t* test. *P < 0.05, **P < 0.01, ***P < 0.001, ****P < 0.0001.

Given that *Shh* was upregulated and Hh signaling was increased after AD induction, we examined directly the impact of Shh in AD, using constitutive Shh^+/–^ mice. On induction of disease, their skin showed significantly lower *Shh* expression than WT mice (Figure 2A). The Shh^+/–^ mice developed aggravated disease compared with WT, with increased epidermal thickness and aggravated hyperkeratosis and parakeratosis (Figure 2B). The Oxa-treated Shh^+/–^ mice showed higher serum IgE, increased *Il4* expression in skin, and increased skin infiltration of CD4^+^ T cells compared with Oxa-treated WT mice (Figure 2, C–E). A greater proportion of skin CD4^+^ T cells expressed IFN-γ and IL-13 in the Shh^+/–^, whereas the proportion of cells that expressed IL-17 was decreased, consistent with faster disease progression (Figure 2F). Thus, the lower level of Shh expression in the skin of Shh^+/–^ mice led to more severe disease.

To confirm the theory that the level of Shh in the skin determines severity of AD, we wanted to induce AD in mice with higher expression of Shh in skin than WT. As Gli3 can reduce *Shh* expression and Shh-signaling (20, 28) we tested if *Shh* expression is increased in skin in constitutive Gli3^+/–^ compared with WT. *Shh* expression was significantly higher in untreated Gli3^+/–^ compared with WT skin (Figure 2G), confirming that this was a suitable mutant to test the impact of elevated levels of Shh on induction of AD. After Oxa treatment, Gli3^+/–^ mice had attenuated epidermal and dermal thickening with decreased hyperkeratosis and parakeratosis compared with WT mice (Figure 2H), and serum IgE levels were significantly decreased (Figure 2I). Significantly lower *Il4* and *Il13* expression and higher *Shh* expression were observed in the Oxa-treated ears of the Gli3^+/–^ group compared with those of the WT mice (Figure 2J). Although we did not detect differences in numbers of skin CD4^+^ and CD8^+^ T cells (Figure 2K), analysis of intracellular cytokine expression showed that the proportion of T cells that expressed the Th2 cytokines IL-4 and IL-13 was decreased in Oxa-treated Gli3^+/–^ compared with Oxa-treated WT mice (Figure 2L). These experiments therefore showed that constitutive Gli3 mutation led to increased *Shh* expression in skin and was protective against induction of AD.

### Pharmacologic blockade of Smoothened caused exacerbation of Oxa-induced AD and Shh downregulation.

**Figure 3:**
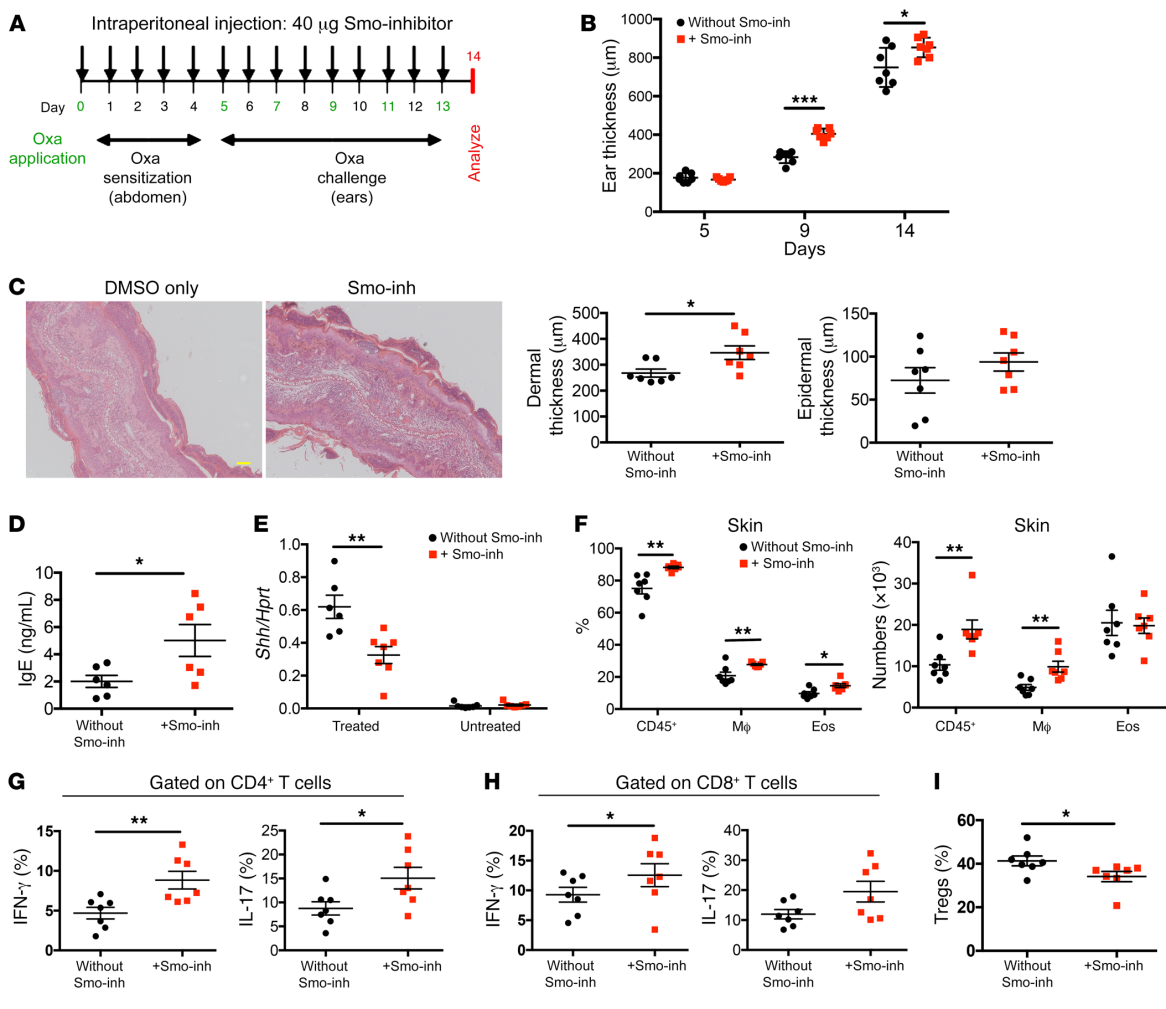
Pharmacological Smo inhibition exacerbates AD in WT mice. Data from 2 independent experiments with 7 Oxa-treated WT mice per group unless otherwise stated. Control (black, DMSO-only–injected) and Smo-inh–injected (red) mice; each symbol represents an individual animal. (**A**) Scheme of Oxa administration with i.p. Smo-inh or DMSO injection. (**B**) Plot shows ear thickness from Oxa-treated control and Smo-inh–injected groups. (**C**) Representative H&E images of ear sections from Oxa-treated WT mice injected either with DMSO only (left image) or Smo-inh (right) at termination (day 14). Scale bar: 100 μm. Plots show dermal and epidermal thickness of Oxa-treated WT without Smo-inh (DMSO-only control) and with Smo-inh on day 14. (**D**) Serum IgE concentration from Oxa-treated control and Smo-inh–injected mice measured by ELISA. (**E**) *Shh* expression (QRT-PCR) in whole ear homogenates from Oxa-treated and Oxa-untreated mice without Smo-inh injection (DMSO-only control) and with Smo-inh injection. (**F**) Percentage and number of skin leukocytes (CD45^+^), macrophages (Mϕ; CD45^+^CD11b^+^F4/80^+^) and eosinophils (Eos; CD45^+^CD11b^+^SiglecF^+^) from ear skin from Oxa-treated control (DMSO only) and Smo-inh–injected mice. (**G**) Percentage of skin CD4^+^ T cells that expressed IFN-γ and IL-17 from Oxa-treated control (DMSO only) and Smo-inh–injected mice. (**H**) Percentage of skin CD8^+^ T cells that expressed IFN-γ and IL-17 from Oxa-treated control (without Smo-inh, DMSO only) and Smo-inh–injected mice. (**I**) Percentage of skin CD4^+^ T cells that are Treg (CD3^+^CD25^+^icFoxp3^+^) from Oxa-treated control (without Smo-inh, DMSO only) and Smo-inh–injected mice. Plots are mean ± SEM; 2-tailed unpaired Student’s *t* test. *P < 0.05, **P < 0.01, and ***P < 0.001.

Given that altering levels of Shh in skin by constitutive genetic mutation changed disease outcome on AD induction, we next investigated in WT mice, whether systemic inhibition of the Hh signaling pathway would also have a detrimental effect on skin pathology on AD induction, using the PF-04449913 Smo-inhibitor (Smo-inh) (29, 30). WT mice received i.p. injections of either Smo-inh or DMSO (vehicle-control) daily throughout the Oxa protocol (Figure 3A). Administration of Smo-inh to Oxa-treated mice caused a significant increase in ear and dermal thickness (Figure 3, B–C) compared with Oxa-treated controls, whereas untreated skin of Smo-inh- and DMSO-injected mice were indistinguishable and contained similar proportions and numbers of skin immune cells (Supplemental Figure 2, A and B). Serum IgE was increased in Oxa-treated Smo-inh–injected mice compared with control mice (Figure 3D), consistent with increased atopic disease. *Shh* transcript levels from Oxa-treated ear tissue were lower in Smo-inh–injected mice compared with control, whereas in the absence of Oxa treatment there was no difference in *Shh* expression in skin of Smo-inh– and DMSO-injected mice (Figure 3E). Smo inhibition significantly increased innate immune cell populations in skin of Oxa-treated mice (Figure 3F). We did not observe significant differences in proportion or numbers of CD4^+^ and CD8^+^ T cells or in their expression of IL-4 and IL-13 (Supplemental Figure 2, C and D), although a significantly greater proportion of CD4^+^ expressed IFN-γ and IL-17 and CD8^+^ T cells expressed IFN-γ (Figure 3, G–H) after Smo-inh administration compared with control. Smo-inh administration led to a significant reduction in the skin CD4^+^CD25^+^Foxp3^+^ Treg population in the Oxa-treated mice (Figure 3I).

Thus, systemic Smo inhibition in WT mice caused increased skin inflammation on induction of AD in vivo and led to reduced *Shh* upregulation in skin tissue, confirming that Shh signaling is important in protecting against AD in WT animals.

### Constitutive inhibition of Hh-mediated transcription in T cells promotes induction of skin inflammation.

**Figure 4:**
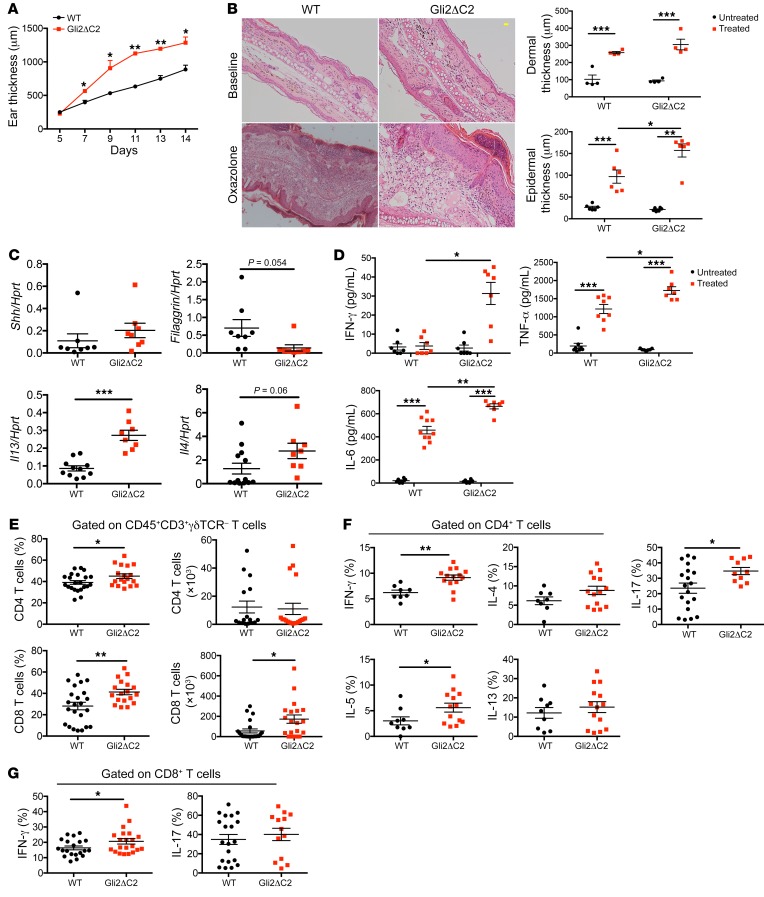
Exacerbated skin inflammation on induction of AD when Hh pathway activation in T cells is inhibited. AD was induced in WT (black) and Gli2ΔC2 (red) mice by Oxa treatment. (**A**) Mean ± SEM ear thickness from Oxa-treated WT and Gli2ΔC2 mice on days 5 to 14 after initiation of Oxa treatment. (**B**) Representative H&E images of skin sections from untreated (baseline) and Oxa-treated WT and Gli2ΔC2 (day 14) mice. Scale bar: 100 μm. Plots show dermal (upper) and epidermal (lower) thickness for control and Oxa-treated groups (day 14); 2-way ANOVA. (**C**) *Shh*, *Filaggrin*, *Il4*, and *Il13* expression (QRT-PCR) in ear homogenates from Oxa-treated WT and Gli2ΔC2 mice. Data from 2 independent experiments. (**D**) Cytokine concentration in skin supernatants from untreated and Oxa-treated WT and Gli2ΔC2 mice. Data from 2 independent experiments, analyzed by 2-way ANOVA. (**E**–**G**) Data from 2 independent experiments with at least 6 mice per group. (**E**) Percentage and number from ear of skin CD4^+^ and CD8^+^ T cells from Oxa-treated WT and Gli2ΔC2 mice. (**F**) Percentage of skin CD4^+^ T cells that express cytokines in Oxa-treated WT and Gli2ΔC2 mice. (**G**) Percentage of skin CD8^+^ T cells that express IFN-γ and IL-17. Two-tailed unpaired Student’s *t* test; 2-way ANOVA (**B**, **D**). Plots are mean ± SEM; each symbol represents an individual animal. *P < 0.05, **P < 0.01, ***P < 0.001.

To investigate the cellular mechanism of the protective role of Hh signaling in AD, we used T cell–specific mutants to test if Shh was signaling directly or indirectly to T cells, since AD is a T cell–dependent disease. We induced AD in mice in which Gli2-mediated transcription is constitutively inhibited in T cells only: lck-Gli2ΔC2 transgenic mice express a truncated form of Gli2 that can function only as a transcriptional repressor, and by binding consensus GBSs inhibits normal Hh-mediated transcription, rendering T cells refractory to upregulation of *Ptch1* on Shh treatment (18). After Oxa treatment, Gli2ΔC2 mice showed significantly increased ear thickness compared with WT mice (Figure 4A). Although nontreated controls of both groups were similar, Oxa-treated Gli2ΔC2 mice had increased dermal and epidermal thickness compared with their Oxa-treated WT littermates (Figure 4B). *Shh* expression in skin was not significantly different, consistent with the fact that the genetic modification in these animals is limited to T cells only, which have not been shown to express Shh (20, 23). In contrast, *Filaggrin* expression was decreased, whereas *Il13* and *Il4* expression were increased in skin from Oxa-treated Gli2ΔC2 mice compared with skin from Oxa-treated WT mice (Figure 4C). Protein levels of IFN-γ, TNF-α, and IL-6, a proinflammatory cytokine secreted by keratinocytes that contributes to AD (31), were higher in Oxa-treated Gli2ΔC2 skin compared with skin from Oxa-treated WT (Figure 4D).

We found significantly higher proportions of CD4 and CD8 T cells and numbers of CD8^+^ T cells in ears from Oxa-treated Gli2ΔC2 mice compared with ears from Oxa-treated WT mice (Figure 4E). In draining lymph nodes (dLNs) there was a significant increase in the proportion of CD8^+^ T cells, and the percentage of CD69^+^ cells was higher in both CD4^+^ and CD8^+^ populations in Oxa-treated Gli2ΔC2 mice compared with Oxa-treated WT mice (Supplemental Figure 3, A and B). Analysis of cytokine production by intracellular staining showed that IFN-γ production was significantly elevated in both Gli2ΔC2 CD4^+^ and CD8^+^ skin populations, and a significantly higher proportion of skin CD4^+^ T cells produced IL-5 and IL-17 compared with WT (Figure 4, F–G). Thus, T cell autonomous inhibition of Hh pathway activation promoted induction of skin inflammation and chronic AD, showing that in normal WT skin, Hh pathway activation in T cells protects against disease induction and severity.

### Gli2-mediated transcription in T cells protects against induction of AD.

**Figure 5:**
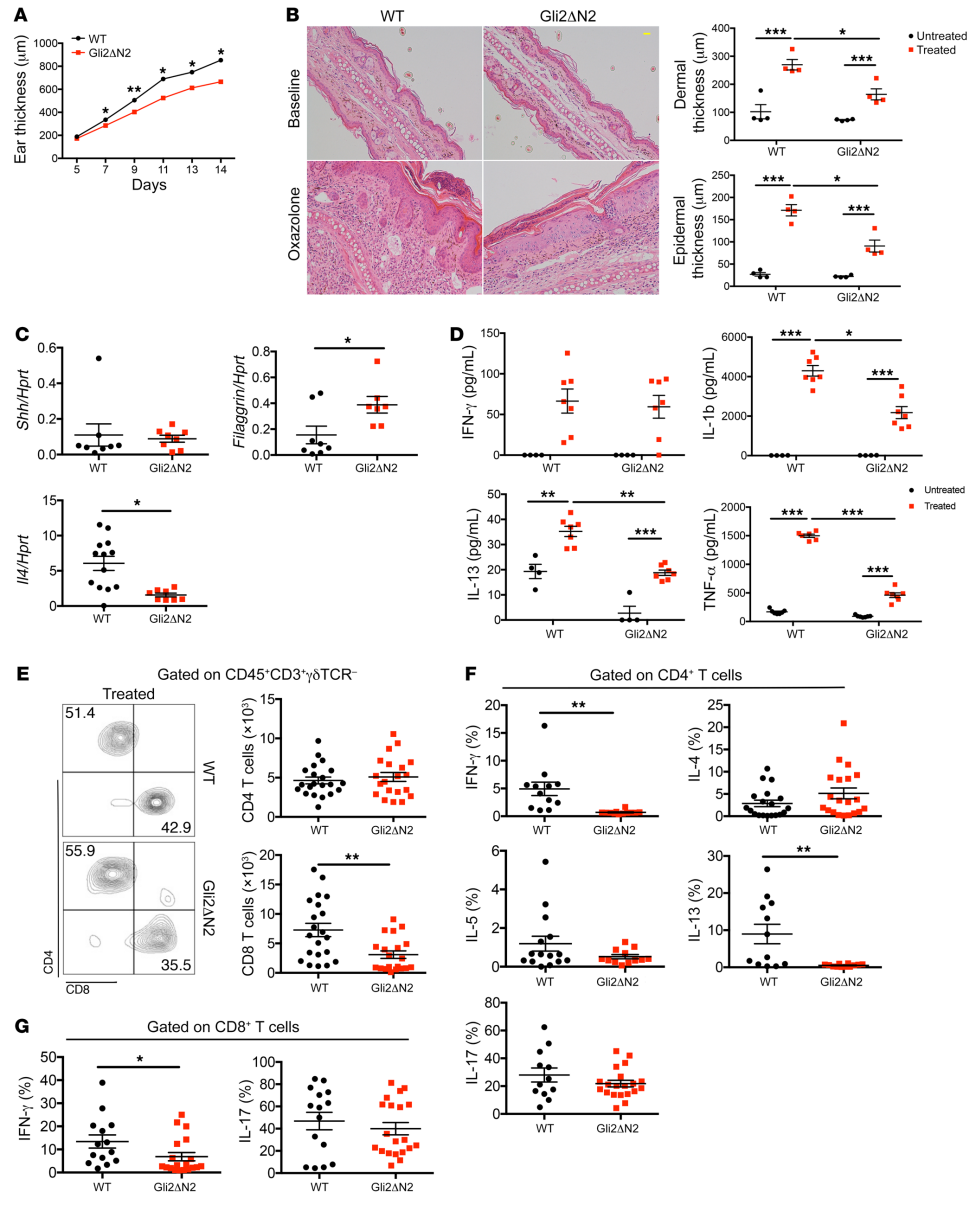
Conditional Gli2-mediated transcription in T cells reduces induction of chronic AD. AD was induced in WT (black) and Gli2ΔN2 (red) mice by Oxa treatment. (**A**) Mean ± SEM ear thickness from Oxa-treated WT (*n* = 8) and Gli2ΔN2 (*n* = 7) mice on days 5 to 14 of Oxa treatment. (**B**) Representative H&E images of skin sections from untreated (baseline) and Oxa-treated WT and Gli2ΔN2 mice on day 14. Scale bar: 100 μm. Plots show dermal and epidermal thickness of untreated and Oxa-treated WT and Gli2ΔN2 mice on day 14. Data from 2 independent experiments; 2-way ANOVA used in **B**. (**C**) *Shh*, *Filaggrin*, and *Il4* expression (QRT-PCR) in ear homogenates from WT and Gli2ΔN2 mice. Data from 2 independent experiments. (**D**) Cytokine concentrations in ear skin supernatants from untreated and Oxa-treated WT and Gli2ΔN2. Data from 2 independent experiments; analysis by 2-way ANOVA. (**E**) Contour plots show CD4 and CD8 expression on skin cells from Oxa-treated WT and Gli2ΔN2 mice. Plots show number of skin CD4^+^ and CD8^+^ T cells isolated from ears. (**F**) Percentages of skin CD4^+^ T cells that express cytokines in Oxa-treated WT and Gli2ΔN2 mice measured by flow cytometry. (**G**) Percentages of skin CD8^+^ T cells that express IFN-γ and IL-17. (**E**–**G**) Data were generated from 2 independent experiments with at least 6 mice per group. Two-tailed unpaired Student’s *t* test; 2-way ANOVA (**B**, **D**). Plots are mean ± SEM; each symbol represents an individual animal. *P < 0.05, **P < 0.01, ***P < 0.001.

To test if elevated Hh pathway activation in T cells can protect against AD, we induced AD in mice in which Gli2-mediated transcription is constitutively activated in T cells only: lck-Gli2ΔN2 transgenic mice express a truncated form of Gli2 that can function only as an activator of transcription, causing constitutive Hh-mediated transcription in all T cells (17). While untreated ears were indistinguishable, Oxa treatment resulted in significantly lower ear thickness from day 7 onward in Gli2ΔN2 mice compared with WT mice, and skin pathology was ameliorated, with diminished dermal and epidermal thickening (Figure 5, A and B). Expression of *Il4* was lower and *Filaggrin* was higher in Oxa-treated Gli2ΔN2 ears compared with WT ears, indicative of better barrier function and lower skin pathology, and we did not detect a difference in *Shh* expression between WT and Gli2ΔN2 groups (Figure 5C). Oxa-treated Gli2ΔN2 ears showed significantly decreased IL-13 protein levels compared with WT ears (Figure 5D). In AD, Th1 cytokines stimulate keratinocytes to produce the proinflammatory cytokines IL-1β and TNF-α, and TNF-α drives IL-1β production by mast cells (32). These proteins were increased in Oxa-treated skin compared with untreated experimental controls, but the Gli2ΔN2 Oxa-treated mice showed significantly lower expression of both cytokines compared with WT mice (Figure 5D). Analysis of T cell skin infiltration did not reveal differences in number of skin CD4^+^ T cells, but there was a significant reduction in skin CD8^+^ T cells in the Oxa-treated Gli2ΔN2 group compared with the WT group (Figure 5E).

Analysis of T cells from dLNs showed significantly reduced CD4^+^ and CD8^+^ populations in Oxa-treated Gli2ΔN2 compared with Oxa-treated WT mice (Supplemental Figure 4A). The proportion of naive (CD62L^+^CD44^–^) CD4^+^ T cells was elevated, whereas the T effector memory (Teff) (CD62L^–^CD44^+^) and T central memory (Tcm) (CD62L^+^CD44^+^) cells were decreased in both CD4^+^ and CD8^+^ populations in dLNs from Oxa-treated Gli2ΔN2 compared with Oxa-treated WT mice (Supplemental Figure 4B), consistent with the fact that Teff and Tcm populations are increased in AD patients (33). Intracellular cytokine analysis showed that in Oxa-treated Gli2ΔN2 skin, a lower proportion of CD4^+^ T cells expressed IFN-γ and IL-13 and a lower proportion of CD8^+^ T cells expressed IFN-γ compared with WT skin, whereas differences in the proportion of cells that expressed IL-4, IL-5, or IL-17 were not statistically significant (Figure 5, F–G).

Thus, conditional Gli2-mediated transcription in T cells impaired induction of AD and rescued skin pathology, showing that elevated T cell autonomous Hh pathway activation can, and is sufficient, to protect against skin inflammation in AD.

### Gli2 controls transcription of inflammation- and immune regulation–related genes.

As the antiinflammatory action of Shh in AD is T cell dependent, we investigated the mechanisms of Shh’s protective role in AD by RNA-sequencing (RNA-seq) FACS-sorted CD4^+^ T cells isolated from the skin of Oxa-treated WT, Gli2ΔN2, and Gli2ΔC2 mice.

**Figure 6:**
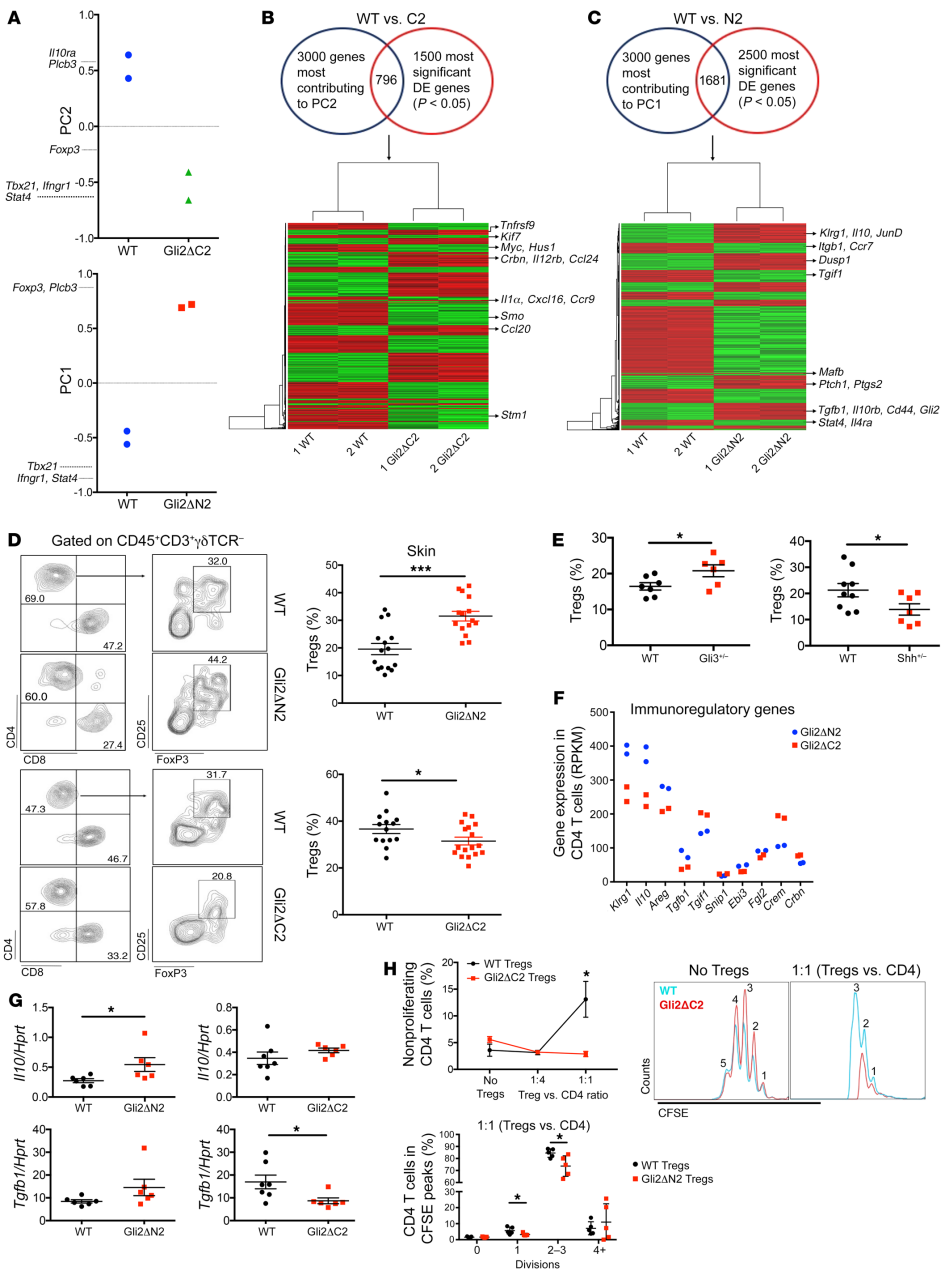
Transcriptional mechanisms of Gli2-mediated antiinflammatory action in skin CD4^+^ T cells. (**A**–**C** and **F**) RNA-seq; data sets from 2 independent experimental groups of FACS-sorted pooled skin CD4^+^ T cells from Oxa-treated WT (*n* = 3), Gli2ΔN2 (*n* = 3), and Gli2ΔC2 (*n* = 3) mice. (**A**) PCA shows PC2 for WT (blue) and Gli2ΔC2 (green) mice; PC1 for WT (blue) and Gli2ΔN2 (red) mice. (**B**) Intersection of 3000 genes that contributed most to PC2 and 1500 most significant DEGs between WT and Gli2ΔC2 mice. (**C**) Intersection of 3000 genes that contributed most to PC1 and 2500 most significant DEGs between WT and Gli2ΔN2 mice. (**B** and **C**) Heatmaps cluster intersection genes; green is lower expression; red is higher expression. (**D**) Representative contour plots show CD4 against CD8 (left); CD25 against Foxp3 (gated on CD4^+^) (right) from Oxa-treated skin from WT and Gli2ΔN2 (top) mice; and WT and Gli2ΔC2 (bottom) mice. Plots show percentage of Tregs (of skin CD4^+^ T cells) in WT (black) or Gli2ΔN2 or Gli2ΔC2 (red). (**E**) Percentage of CD25^+^Foxp3^+^, gated on skin CD4^+^ from Oxa-treated littermates. Left: WT (black), Gli3^+/–^ (red). Right: WT (black), Shh^+/–^ (red). (**F**) Expression of immune regulation genes from Gli2ΔN2 (blue) and Gli2ΔC2 (red) RNA-seq. (**G**) *Il10* and *Tgfb1* expression (QRT-PCR) in Oxa-treated ear homogenates from littermates: WT (black) and Gli2ΔN2 (red) (left); WT (black) and Gli2ΔC2 (red) (right); 2 independent experiments. (**H**) Two independent experiments. Top: WT-CD4^+^CD25^–^ T cells cocultured with CD4^+^CD25^+^ cells from Oxa-treated Gli2ΔC2 and WT spleens and WT-CD4^+^CD25^–^ proliferation (CFSE staining). Plot shows percentage of nonproliferating cells on addition of WT-Tregs (black) and Gli2ΔC2-Tregs (red). Bottom: WT-CD4^+^CD25^–^ T cells cocultured (1:1) with CD4^+^CD25^+^ cells from Oxa-treated Gli2ΔN2 and WT spleens. Plot shows percentage of cells that underwent divisions (CFSE staining) for WT-Tregs (black) and Gli2ΔN2-Tregs (red). Representative histograms show cell divisions without Tregs (left) and divisions cocultured 1:1 (right) with WT-Tregs (red) and Gli2ΔC2-Tregs (blue). Plots show mean ± SEM; for **D**–**E**, **G**, and **H** (bottom) each symbol represents an individual animal; 2-tailed unpaired Student’s *t* test. *P < 0.05, ***P < 0.001.

First, to evaluate the RNA-seq data sets in an unbiased way, we carried out principal component analysis (PCA), a multivariate analysis, which can be used to segregate genome-wide transcription data sets according to variability in transcript expression values and cluster data sets to detect dominant patterns of gene expression, represented by the principal components (PC). PCA segregated our data sets by genotype on PC1 and PC2. PC1 accounted for 33% of variability and separated WT from Gli2ΔN2, whereas PC2 accounted for 20% of variability and separated WT from Gli2ΔC2 (Figure 6A). Genes that contributed strongly to these axes included *Stat4*, *Tbx21*, and *Ifngr1*, which were upregulated in samples from Gli2ΔC2 mice (represented by negative scores in PC2 axis) and downregulated in samples from Gli2ΔN2 mice (negative scores in PC1 axis). Additionally, *Foxp3*, *Il10ra*, and *Plcb3* contributed strongly to PC1 and PC2, and showed increased transcription in Gli2ΔN2 mice compared with WT mice, and decreased transcription in Gli2ΔC2 mice compared with WT mice. Thus, PCA highlighted differences in expression of genes involved in inflammatory T cell responses and immune regulation.

To further investigate the genes that are implicated in functional differences between genotypes in chronic AD, we intersected differentially expressed genes (DEGs) identified by Ebayes statistics between WT and each transgenic sample with genes that contributed to the relevant PC axis for that transgenic sample. For the comparison between Gli2ΔC2 and WT mice, 796 DEGs from the intersection (Supplemental Table 1) were clustered in a heatmap (Figure 6B). Hh pathway and target genes (*Hus1*, *Stmn1*, *Kif7*, *Smo*) (9, 28, 34, 35) were downregulated in Gli2ΔC2 compared with WT mice, whereas genes involved in inflammation (*Tnfrsf9*, *Il1a*, *Il12rb*), migration (*Ccr9*, *Cxcl16*, *Ccl20*, *Ccl24*), and inhibition of immune regulation (*Crbn*) (36) were upregulated in Gli2ΔC2 compared with WT mice.

For the comparison between Gli2ΔN2 and WT mice, 1681 DEGs from the intersection genes (Supplemental Table 2) were clustered in a heatmap (Figure 6C). Hh pathway components, target genes (*Ptch1*, *Gli2*, *Mafb*) (9, 10, 34), and immune-regulatory genes (*Tgfb1*, *Il10*, *Il10rb*, *Klrg1*, *Cd44*) (6) were upregulated in Gli2ΔN2 compared with WT samples, whereas *Tgif1*, a negative regulator of TGF-β (37), inflammatory (*Stat4*, *Il4ra*), and migratory (*Ccr7*) genes were downregulated in the Gli2ΔN2 data sets compared with WT samples. *Ptgs2*, which is both a Hh target gene and a positive regulator of Tregs (38), was upregulated in Gli2ΔN2 samples.

*Shh* and *Gli3* expression was not detected in skin CD4^+^ T cells in any data set (Supplemental Table 3), consistent with previous studies showing *Shh* and *Gli3* expression in epithelial cells but not mouse T cells (15, 20, 23, 39–41).

Thus, on induction of AD, elevated levels of Hh-mediated transcription in skin CD4^+^ T cells promoted expression of immune-regulatory genes and repressed inflammation genes, whereas reduction of Hh-mediated transcription in T cells to below WT levels promoted expression of inflammation genes.

### Hedgehog signaling is required for activation of skin Treg populations.

As the RNA-seq revealed involvement of immune-regulatory genes, we tested if Hh signaling influences skin and peripheral Treg populations after AD induction. After Oxa treatment, we observed a significant increase in CD4^+^CD25^+^Foxp3^+^ (Tregs) in Gli2ΔN2 and Gli3^+/–^ skin compared with WT skin, and a significant reduction in the proportion of Tregs in Gli2ΔC2 and Shh^+/–^ skin compared with WT skin (Figure 6, D and E). Likewise, Tregs in dLNs from Oxa-treated Gli2ΔC2 mice were significantly decreased compared with WT mice (Supplemental Figure 5A).

DEGs between Gli2ΔN2 and Gli2ΔC2 data sets included genes involved in Treg function (*Klrg1*, *Il10*, *Tgfb1*, and *Areg*) which were significantly upregulated in Gli2ΔN2 compared with Gli2ΔC2 samples, whereas genes that negatively regulate TGF-β signaling or Treg function (*Tgif1*, *Crem*, *Crbn*) were more highly expressed in the Gli2ΔC2 data sets (Figure 6F).

The Tregs in Oxa-treated Gli2ΔN2 dLNs displayed a more activated and suppressive phenotype than their WT counterparts, with increased expression of Klrg1, CTLA4, CD44, and Ki67, whereas inhibition of normal Hh-mediated transcription in Oxa-treated Gli2ΔC2 dLNs led to a significant decrease in Klrg1, CTLA4, and CD44 expression on Tregs, compared with WT dLNs (Supplemental Figure 5, B and C).

*Il10* and *Tgfb1* were differentially expressed between genotypes in CD4^+^ T cells isolated from Oxa-treated skin (Figure 6F), and as these genes are also expressed by other immune cell types, we evaluated their expression in whole skin (ear tissue). Oxa-treated Gli2ΔN2 mice showed higher *Il10* expression in ear tissue than WT mice, whereas Oxa-treated Gli2ΔC2 mice showed lower *Tgfb1* expression in ear tissue than WT mice (Figure 6G), indicating that inhibition of Hh-mediated transcription specifically in T cells is sufficient to reduce overall expression of the gene encoding this important antiinflammatory cytokine in the skin.

Given that Tregs from Gli2ΔN2 mice had a stronger immunoregulatory phenotype than Tregs from WT or Gli2ΔC2 mice (Figure 6, A–C and F, and Supplemental Figure 5, B and C) we compared their in vitro immunosuppressive activity in coculture with WT cells (Figure 6H). Tregs isolated from spleen of Oxa-treated Gli2ΔC2 mice had lower in vitro immunosuppressive activity than Tregs from Oxa-treated WT mice, so that not only was the phenotypically defined active Treg population reduced, but its function was compromised by inhibition of Gli-mediated transcription (Figure 6H). In contrast, Tregs isolated from spleen of Oxa-treated Gli2ΔN2 mice had higher immunosuppressive activity than Tregs from Oxa-treated WT mice (Figure 6H), consistent with their more activated phenotype (Figure 6, C and F, and Supplemental Figure 5, B and C).

**Figure 7:**
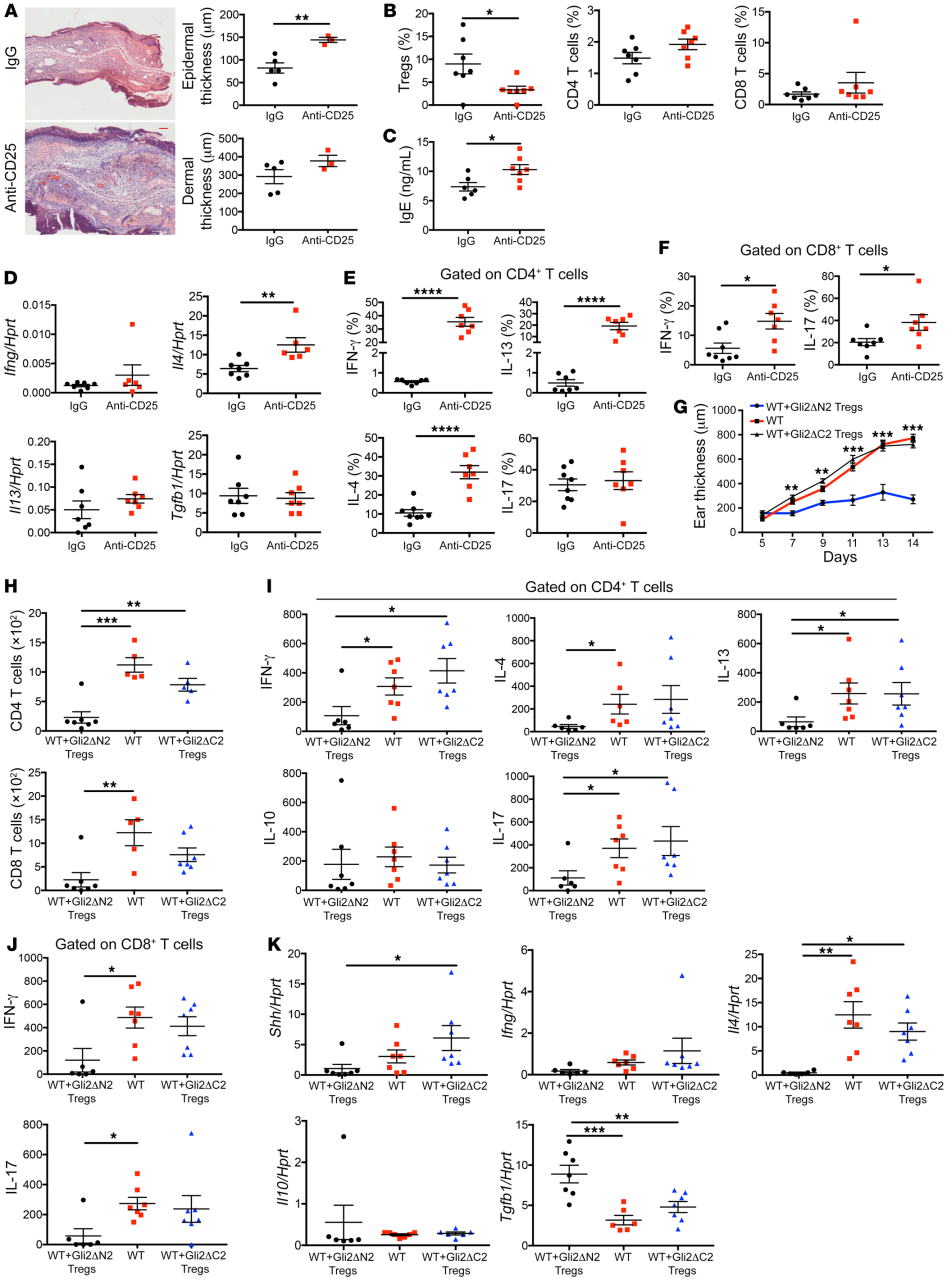
Hh signaling promotes skin Treg populations and their immune-regulatory function. (**A**–**F**) Groups of Gli2ΔN2 mice (7 per group unless stated otherwise) were injected in vivo with anti-CD25 antibody (red) or control IgG (black) 4 days before initiation of the Oxa protocol and again on day 7 (Supplemental Figure 5D). (**A**) Representative H&E images of skin sections from Oxa-treated anti-CD25–injected (*n* = 3) and control (IgG-injected, *n* = 5) Gli2ΔN2 groups on day 14. Scale bar: 100 μm. Plots show dermal and epidermal thickness. (**B**) Percentage of Tregs (CD25^+^icFoxp3^+^) (gated on CD4^+^) from ears of control and anti-CD25–treated groups (left). Percentage of CD4^+^T cells and CD8^+^T cells (gated on live cells) from ears (center and right). (**C**) Serum IgE concentration (ELISA) from control and anti-CD25–treated groups. (**D**) *Ifng*, *Il4*, *Il13*, and *Tgfb1* expression (QRT-PCR) in ear homogenates. (**E**) Percentage of skin CD4^+^ T cells that express cytokines. (**F**) Percentage of skin CD8^+^ T cells that express cytokines. (**G**–**K**) Adoptive transfer into Oxa-treated WT mice. Purified CD4^+^CD25^+^ (Tregs) from spleens of Oxa-treated Gli2ΔN2 (Gli2ΔN2 adoptive transfer group, *n* = 7, black) or Gli2ΔC2 mice (Gli2ΔC2 adoptive transfer group, *n* = 7, blue) were injected into WT mice 2 days before and on day 7 of Oxa protocol (Supplemental Figure 5E), and compared with Oxa protocol WT control mice (control group; *n* = 7, red). (**G**) Ear thickness on days 5 to 14 of the Oxa protocol of WT control, Gli2ΔN2 adoptive transfer, and Gli2ΔC2 adoptive transfer groups (**P < 0.01 and ***P < 0.001, 2-tailed *t* test between WT control and Gli2ΔN2 adoptive transfer group). (**H**) Number of CD4^+^ T cells and CD8^+^ T cells recovered from ears from the 3 experimental groups. (**I**) Number of CD4^+^ T cells that expressed cytokines. (**J**) Number of CD8^+^ T cells that expressed cytokines. (**K**) *Ifng*, *Il4*, *Il10*, *Tgfb1*, and *Shh* expression (QRT-PCR) in ear homogenates. Two-tailed unpaired Student’s *t* test. (**I**–**K**) One-way ANOVA; each symbol represents an individual animal, showing mean ± SEM. *P < 0.05, **P < 0.01, ***P < 0.001, ****P < 0.0001.

To test in vivo the importance of Hh-driven Treg function in AD induction and progression, we depleted Tregs from Gli2ΔN2 mice by anti-CD25 mAb injection before and during the Oxa protocol (Supplemental Figure 5D) and compared the outcome to control Oxa-treated Gli2ΔN2 mice that were injected with irrelevant IgG (Figure 7, A–F). Anti-CD25 injection significantly reduced the Treg population and led to increases in ear swelling and epidermal thickness, serum IgE, *Il4* expression in ear skin, and the proportion of skin CD4^+^ T cells that expressed intracellular IFN-γ, IL-13, and IL-4 (Figure 7, A–E). The proportion of skin CD8^+^ T cells that expressed intracellular IFN-γ and IL-17 were also increased in the anti-CD25 group compared with the control group (Figure 7F). Depletion of the Treg population was therefore sufficient to increase induction of AD and inflammation on Oxa treatment in Gli2ΔN2 mice.

Next, to test the importance of Hh signaling in induction of Treg immunoregulatory function in AD in vivo, we compared the ability of CD4^+^CD25^+^ (Treg) cells purified from Oxa-treated Gli2ΔN2 or Gli2ΔC2 mice to inhibit AD induction and reduce disease severity and inflammation on adoptive transfer into Oxa-treated WT recipients (Figure 7, G–K). Purified Tregs were injected into the recipients 2 days before initiation of the Oxa protocol and again on day 7 (Supplemental Figure 5E). Adoptive transfer of Gli2ΔN2 Tregs significantly reduced ear thickness compared with Oxa-treated WT control from day 7 of the Oxa protocol onwards, whereas adoptive transfer of Gli2ΔC2 Tregs had no impact on ear thickness (Figure 7G). The number of CD4^+^ and CD8^+^ T cells in Oxa-treated recipient ears was reduced by approximately 4-fold in the Gli2ΔN2 adoptive transfer group compared with the control group, but was not significantly different between the Gli2ΔC2 adoptive transfer group and the control group (Figure 7H). The proportion of IL-17^+^ cells in the skin CD4^+^ population was significantly reduced in the Gli2ΔN2 adoptive transfer group compared with the control group (Supplemental Figure 5F), and the number of CD4^+^ cells isolated from Oxa-treated ears that expressed IFN-γ, IL-13, IL-4, and IL-17 and the number of CD8^+^ cells that expressed IFN-γ and IL-17 were also significantly reduced in the Gli2ΔN2 adoptive transfer group, but were not different between Gli2ΔC2 adoptive transfer group compared with the control group (Figure 7, I–J). Whole ear tissue showed significantly lower expression of *Il4* and higher expression of *Tgfb1* in the Gli2ΔN2 adoptive transfer group compared with the control group, but expression of these genes was not significantly different between the Gli2ΔC2 adoptive transfer group and the control group (Figure 7K). Interestingly, *Shh* expression was also significantly reduced in the Gli2ΔN2 adoptive transfer group compared with the Gli2ΔC2 adoptive transfer group (Figure 7K), indicating that the T cell autonomous immunoregulatory action of Hh signaling to induce Treg activity is sufficient to inhibit Shh upregulation in response to Oxa-induced inflammation.

### Hh pathway activation in T cells activates TGF-β signaling for immune regulation.

**Figure 8:**
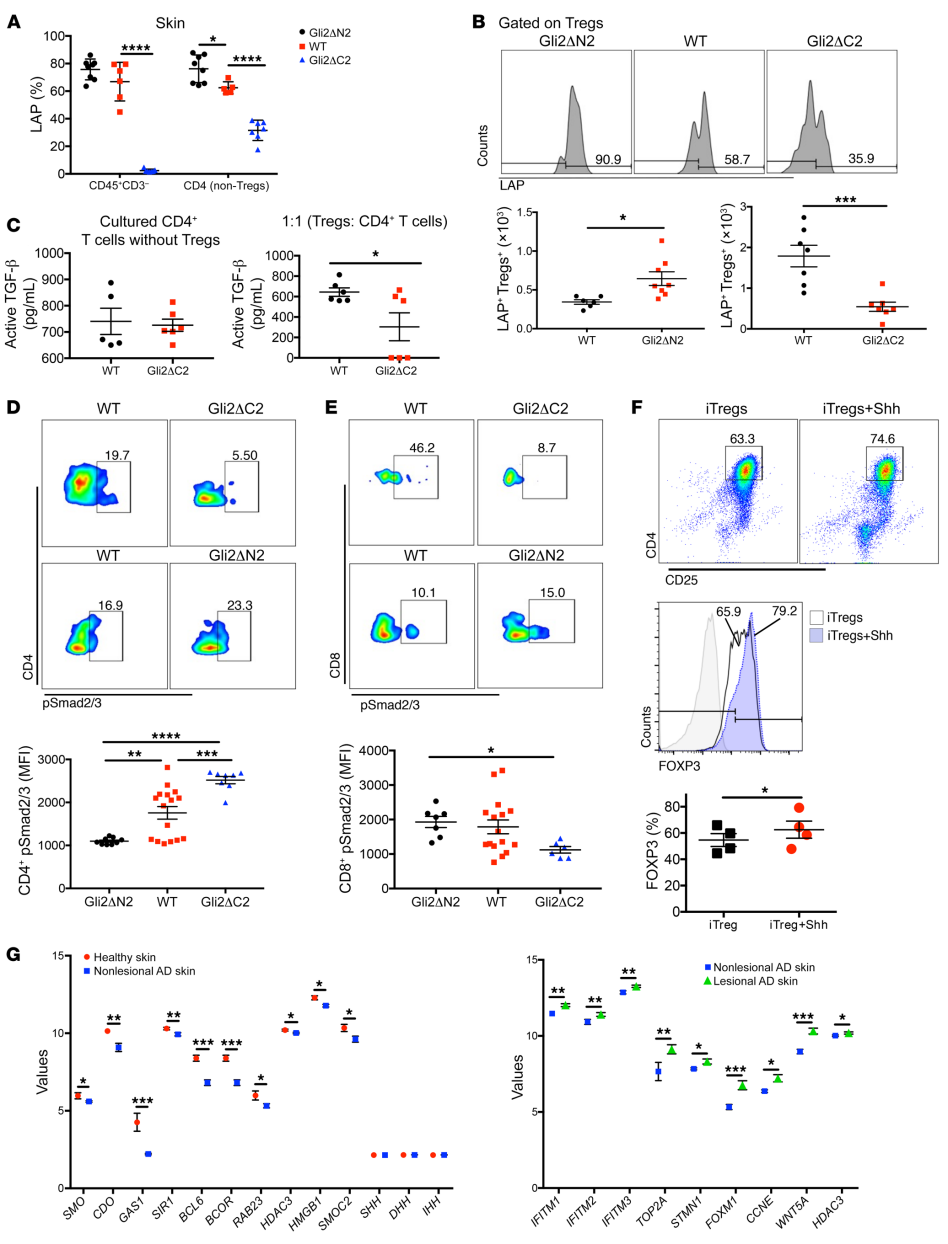
Hh signaling promotes immune-regulatory function through TGF-β. Hedgehog signaling and human AD. (**A**–**E**) Two independent experiments with at least 6 mice per group. (**A**) Percentage of LAP^+^ cells, gated on CD45^+^CD3^–^ and CD4^+^CD25^–^ (non-Treg) from skin of Oxa-treated Gli2ΔN2 (black), WT (red), and Gli2ΔC2 (blue) mice. (**B**) Representative histograms show anti-LAP staining on skin Tregs from Oxa-treated littermates. Plots show number of LAP^+^ Tregs in WT (black) littermates and Gli2ΔN2 (red) (left) or Gli2ΔC2 (red) (right). (**C**) Active TGF-β concentration (ELISA) in culture supernatants. CD4^+^CD25^–^ splenocytes from WT (black) and Gli2ΔC2 (red) mice, cultured alone (left). WT CD4^+^ T cells cultured 1:1 with FACS-sorted Tregs (CD4^+^CD25^+^) from Oxa-treated WT (black) and Gli2ΔC2 (red) mice (right). (**D** and **E**) Representative FACS plots show pSmad2/3 expression in skin (**D**) CD4^+^ and (**E**) CD8^+^ (gated on CD45^+^CD3^+^γδTCR^–^) from Oxa-treated WT and Gli2ΔC2 mice (left); and WT and Gli2ΔN2 mice (right). Plots show MFI of anti-pSmad2/3 in skin CD4^+^ (left) and CD8^+^ (right) from Gli2ΔN2 (black), WT (red), and Gli2ΔC2 (blue) Oxa-treated groups (1-way ANOVA). (**F**) Naive CD4^+^ cells from 4 randomly selected unknown leukocyte-cone donors cultured for 5 days under iTreg conditions in presence or absence of rShh (labeled iTregs). Dot-plot shows representative CD4 and CD25 expression. Histogram shows representative icFOXP3 expression, gated on CD4^+^CD25^+^ and giving percentage of cells in marker shown. Scatter plot shows percentage of FOXP3^+^ cells gated on CD4^+^CD25^+^ from cultures from 4 independent donors; 2-tailed paired *t* test. (**G**) Scatter plots show expression (RPKM) from publicly available RNA-seq data sets (GEO GSE32924) from human healthy skin (red, *n* = 8), and nonlesional (blue) and lesional (green) skin from AD patients (*n* = 12). Expression of genes encoding Hh pathway components and regulators in healthy skin and nonlesional skin from AD patients (left). Expression of Hh target genes in nonlesional and lesional skin from AD patients (2-tailed paired *t* test) (right). Two-tailed unpaired Student’s *t* test unless stated otherwise; plots are mean ± SEM, each symbol represents a different individual. *P < 0.05, **P < 0.01, ***P < 0.001, ****P < 0.0001.

The adoptive transfer experiments showed both that Gli2-mediated transcription in T cells is essential for Treg immune-regulatory function and that Hh-driven immune-regulatory function is sufficient to inhibit AD induction (Figure 7, G–K). In addition, *Tgfb1* was differentially expressed in RNA-seq data sets and in whole ear between Oxa-treated Gli2ΔN2 and Gli2ΔC2 mice (Figure 6, F–G). Therefore, as TGF-β signals for induction, maintenance, and function of Tregs (42, 43), we evaluated TGF-β by staining against anti–latency associated peptide (anti-LAP) in skin populations from Oxa-treated Gli2ΔN2, Gli2ΔC2, and WT mice. LAP is derived from cleavage of the N-terminal of the TGF-β precursor protein and held on the cell surface, and its activation is crucial for the suppressive potential of Tregs and TGF-β function (44, 45). Non-Treg CD4^+^ T cells from Oxa-treated Gli2ΔN2 mice expressed significantly more LAP than WT mice, whereas the Oxa-treated Gli2ΔC2 mice showed a reduction in cell-surface LAP on CD45^+^ cells and non-Treg CD4^+^ T cells compared with WT mice (Figure 8A). Expression of LAP was increased on the Oxa-treated Gli2ΔN2 Treg population compared with WT Tregs, but significantly reduced on the Gli2ΔC2 Treg population compared with WT Tregs (Figure 8B). We then measured the concentration of active TGF-β protein in supernatants from in vitro immunosuppressive assays between WT and Gli2ΔC2 Tregs (Figure 8C). WT CD4–Gli2ΔC2 Treg cocultures contained significantly less active TGF-β compared with WT CD4–WT Treg cocultures, consistent with the anti-LAP staining, RNA-seq expression profiles, and disease severity upon Oxa treatment when Gli2-mediated transcription in T cells is inhibited.

Previous studies showed that Tregs fail to suppress T cells, which have a reduced ability to respond to TGF-β in mouse models of colitis and autoimmune encephalomyelitis (46–48). Given the reduced LAP expression, dampened TGF-β, and compromised cell-mediated suppression of Gli2ΔC2 Tregs in vitro and in vivo, we measured TGF-β signal transduction in skin Teff populations by staining against pSmad2/3. Skin CD4^+^ and CD8^+^ T cells from Oxa-treated Gli2ΔC2 mice showed decreased pSmad2/3 expression compared with WT mice, whereas skin CD4^+^ and CD8^+^ T cells from Oxa-treated Gli2ΔN2 mice had increased pSmad2/3 expression compared with WT mice (Figure 8, D and E), indicating that TGF-β production requires Hh-dependent Treg function in chronic AD and TGF-β signals directly to skin Teffs.

Taken together, our data demonstrate that Hh signaling induces and maintains functional Treg populations in the skin, leading to upregulation and activation of TGF-β, thus identifying a mechanism by which Gli2 activity in CD4^+^ T cells can control skin-specific immune responses and inflammation and reduce chronic AD.

### Shh induced human Treg differentiation in vitro.

Given the protective function of Shh and Hh signaling in our in-bred mouse model, we wanted to extrapolate our study to human AD. As Hh pathway activation induced Treg differentiation and Foxp3 expression in mice (Figure 6, A–F), we tested if this is also the case in human T cells in vitro. We purified naive human CD4^+^ T cells from 4 independent randomly selected unknown donors and cultured them for 5 days under iTreg conditions (plate-bound anti-CD3, soluble anti-CD28, IL-2, and TGF-β1) in the presence of rShh or without addition of rShh (control). Gating on CD4^+^CD25^+^, the addition of rShh increased the expression of FOXP3 in cultures from all 4 independent donors (Figure 8F), suggesting that Shh can promote Treg differentiation in human T cells.

### Expression of Hh pathway components in human skin from healthy donors and AD patients.

As Shh treatment increased human Treg differentiation in vitro, and in mice, decreasing Hh levels in Shh^+/–^ skin increased AD severity, but increasing Shh expression in Gli3^+/–^ skin decreased AD severity, we hypothesized that individuals who suffer from AD might have lower baseline levels of expression of Hh pathway components in skin than healthy individuals. To test this, we compared levels of expression of Hh pathway components in transcriptome data sets from healthy donors and from healthy (nonlesional) skin from individuals who suffer from AD (GEO GSE32924) (49). Although we found no significant differences in expression of *SHH*, *IHH*, or *DHH*, mean expression levels of key components of the pathway, including the nonredundant signal transducer *SMO* and the Hh coreceptors *CDO* and *GAS1*, were lower in nonlesional skin from AD patients than in skin from healthy controls (Figure 8G), suggesting that baseline levels of Hh signaling are indeed lower in humans who suffer from AD. Mean expression of several other genes known to interact with the pathway or are SHH target genes were also lower in nonlesional skin from AD patients than in skin from healthy controls (Figure 8G) (28, 50–53). We then compared gene expression in lesional skin from AD patients to healthy (nonlesional) skin from the same individuals. Expression of several known SHH target genes (20, 24, 34, 54, 55) was increased in lesional skin compared with nonlesional skin from the same individuals (Figure 8G), suggestive of increased activation of the pathway in inflamed skin.

## Discussion

Here we demonstrate that Shh signaling regulates the skin T cell immune response in AD. Cross-talk between epithelial cells and lymphocytes is essential for maintenance of skin homeostasis, and disruption of skin barrier function together with immune dysregulation lead to AD. Our experiments show that in the skin the morphogen Shh signals to CD4^+^ T cells to induce active regulatory T cell function and prevent inflammation, protecting against AD.

We showed that Shh was upregulated in skin on induction of AD and that this was protective against disease pathology and skin inflammation. The levels of Shh expression determined disease outcome. When Shh expression was lowered below WT levels in Shh^+/–^ mice, AD was more severe, but when Shh was increased to above WT levels in Gli3^+/–^ mice, AD was ameliorated. Thus, in humans, genetic factors that determine degree of Hh pathway activation in skin are likely to influence susceptibility to AD, and Hh-activating molecules might offer a new therapeutic approach. In support of this, analysis of human transcriptome data sets showed that baseline levels of key components of the Hh signaling pathway were expressed at lower levels in nonlesional skin of AD sufferers than in healthy control skin, indicating that Hh signaling may also be protective in human AD. In addition, genome wide association (GWAS) studies have identified the kinesin family member *KIF3A* as a susceptibility gene for AD (56, 57). KIF3A is required for translocation of SMO in the primary cilium on SHH signal transduction (10), so its identification in GWAS further supports the involvement of Hh signaling in human AD.

AD is a T cell–dependent disease and we showed that the antiinflammatory action of Shh was orchestrated by T cells, as when normal Hh pathway activation was conditionally inhibited in T cells all parameters of disease progression, severity, and pathology were increased. Hh-mediated transcription in response to Shh upregulation increased skin CD4^+^ Treg function, activated TGF-β production, and promoted TGF-β signaling to other skin T cells, thereby decreasing skin proinflammatory responses. This was supported by in vivo experiments that showed that adoptive transfer of Tregs from Oxa-treated Gli2ΔN2 animals was sufficient to prevent AD in Oxa-treated WT animals, whereas adoptive transfer of Tregs in which the Hh signaling pathway is inhibited (Oxa-treated Gli2ΔC2) was unable to reduce AD, highlighting the essential role of Hh pathway activation for immune regulation on induction of AD.

RNA-seq demonstrated that Hh-mediated transcription in skin CD4^+^ T cells on induction of AD increased expression of immunoregulatory genes (including *Ptgs2*, *Il10*, *Klrg1*, *Areg*, and *Fgl2*) and decreased expression of inflammatory and migratory genes (*Tnfrsf9*, *Il1a*, *Tgif1*, *Ccr7*, *Ccr9*, *Ccl20*, and *Ccl24*). The observation that increased Hh pathway activation restores the immunosuppressive phenotype of skin Tregs is important for treatments for AD, because nonfunctional Tregs are strongly correlated with skin inflammation in mouse models and human patients (5–8, 58).

Notch and Wnt signaling are also involved in skin inflammation. Loss of Notch signaling results in disruption of the epidermal skin barrier and inflammatory skin disease (59, 60), whereas the Wnt pathway can drive skin inflammatory responses and is induced in psoriasis (61–63). Genes encoding components of Wnt (*Tcf4*, *Wnt4*) and Notch pathways (*Dtx1*, *Hes*, *Dtx3*, *Lfng*) were differentially expressed between genotypes in our RNA-seq data sets, suggesting crosstalk between Hh signaling and these other developmental pathways in skin T cells.

In contrast to the demonstrated protective role of Shh upregulation on induction of chronic AD, increased Shh expression and Hh pathway activation in T cells in the lung exacerbates allergic asthma by promoting Th2 differentiation (23, 25). The fact that Shh is a morphogen that signals for distinct functions dependent on strength and duration of signal might account for its different roles in AD and asthma, and our experiments highlight the context dependency of Shh’s functions and the tissue specificity of immune regulation by CD4^+^ T cells. An antiinflammatory action of Hh signaling has been described in other tissues (64–70), and here we demonstrate the beneficial consequences of Shh upregulation in AD. Additionally, we show that the antiinflammatory action of Shh in AD is orchestrated by T cells, and dissect the T cell intrinsic mechanisms of protection.

Aberrant Hh protein expression or dysregulated Hh signaling is involved in epithelial-derived tumors, including BCC (12, 13). We showed that inhibition of the Hh pathway both by pharmacological blockade and by genetic T cell–specific inhibition of endogenous Hh-mediated transcription increased IFN-γ production in skin CD4^+^ and CD8^+^ T cells. This is consistent with a recent study in which pharmacological Hh pathway inhibition resulted in BCC regression and a concomitant increase in IFN-γ^+^CD8^+^ infiltration (71). As Shh signals to induce regulatory functions in skin T cells, our study indicates that Hh secretion might be a mechanism of immune evasion by skin tumors. Inhibition of Shh-signaling in skin cancers will therefore have the additional benefit of releasing T cells to generate an immune response against the tumor.

As well as highlighting that Hh-mediated transcription in T cells promotes TGF-β signaling to dampen skin inflammation, our RNA-seq of CD4^+^ T cells from AD skin showed that Hh reduced expression of *Ccl24* and *Ccl8*, and these chemokines recruit eosinophils to the skin (72). Hh also reduced expression of other genes for inflammatory signaling pathways implicated in skin inflammation including TNFR (*Tnfrsf25*), JAK-STAT (*Jak1*), and NF-κB (*Trl4*, *Myd88*, *Cd69*, *Cd48*, *Irf1*).

Research into therapies for AD has focused on targeting Th2 cytokines (73). The observation that Shh is upregulated in skin after AD induction and that Gli-dependent Hh pathway activation in T cells suppresses proinflammatory cytokine production and restores functional Treg populations, is important for development of new strategies to treat AD.

## Methods

### Mice and atopic dermatitis model by chronic hapten application.

GBS-GFP transgenic mice (27) were provided by J. Briscoe (Francis Crick Institute, London, United Kingdom) and Shh^+/–^ mice were provided by P. Beachy (Stanford University, Stanford, California, USA) (74). C57BL/6 mice were purchased from Envigo; Gli3^+/–^ and Rag1-KO mice were from the Jackson Laboratory. Lck-Gli2ΔN2 and Lck-Gli2ΔC2 transgenic mice were as described (17, 18). Mice were fully backcrossed onto C57/BL6 and maintained in specific pathogen-free conditions at UCL.

Chronic AD was induced as described (75). Six- to 8-week-old mice were sensitized on the shaved abdomen with 3% of the hapten 4-ethoxymethylene-2-phenyloxazolin-5-one (Oxa, Sigma) and challenged topically on the ears with 5 doses of 0.6% Oxa on days 5, 7, 9, 11, and 13. Mice were sacrificed and analyzed on day 14 (Figure 1A). For controls, vehicle (ethanol) alone was applied. This protocol was used in all experiments and is referred to as Oxa treatment. All analyses were made on termination on day 14, unless otherwise stated. Ear thickness was measured with a micrometer (Mitutoyo).

In some experiments, mice were injected i.p. with 40 μg/day of Smo-inh, PF-04449913 (Pfizer), or vehicle control (DMSO) for 14 days (Figure 3A).

### Tissue processing and flow cytometry.

Spleens and skin-draining LNs were processed and stained as described (35), using antibodies listed in Supplemental Table 4. Cell counting was performed on an Accuri C6 (BD Biosciences) and/or hemocytometer. To release leukocytes from ear tissue, ears were split into dorsal and ventral halves and digested for 30 minutes at 37°C with 1 mg/mL Collagenase D (Roche) and 0.5 mg/mL DNase I (Roche) in RPMI (Life Technologies) containing 2%FCS (Life Technologies) and 2% penicillin/streptomycin (Life Technologies), filtered through a 70-μm cell strainer (VWR) and washed in RPMI containing 10% FCS and 5% penicillin/streptomycin. Intracellular cytokine staining was performed after restimulation of cells in complete RPMI (cRPMI) with cell activation cocktail (Biolegend) for 4 hours at 37°C. Cells were first surface stained, fixed/permeabilized using the eBioscience Fixation/Permeabilization kit according to the manufacturer’s instructions, and then stained for cytokines.

For pSmad2/3 staining, cells were stimulated for 45 minutes with rTGF-β (5 ng/mL) in RPMI at 37°C and 5% CO_2_, then fixed with BD PhosphoFlow PermBufferII according to the manufacturer’s instructions, washed with PBS, and permeabilized with ice-cold BD PhosphoFlow PermBufferIII for 30 minutes according to the manufacturer’s instructions. To block unspecific binding, cells were treated with 1.5% donkey serum (MilliporeSigma) in PBS for 30 minutes. After washing with PBS, cells were incubated with primary anti-Smad2/3 antibody (1:50) for 1 hour before incubation with donkey anti-rabbit–PE secondary antibody (1:200) and other antibodies of interest for 45 minutes.

Dead cells were excluded using the Zombie Aqua Fixable Viability kit (Biolegend). Cells were analyzed on LSRII (BD Biosciences). Data were analyzed using FlowJo 10.4.1 (Tree Star).

### Treg neutralization in vivo and Treg adoptive transfer.

For Treg neutralization, Tregs from Lck-Gli2ΔN2 mice were depleted in vivo using 100 μg anti-CD25 antibody (PC61, Biolegend) and control Lck-Gli2ΔN2 mice were injected with 100 μg rat IgG (Biolegend) 4 days before the Oxa treatment and on day 7 (Supplemental Figure 5D).

For adoptive transfer experiments, Gli2ΔN2 and Gli2ΔC2 Tregs from spleens of Oxa-treated mice were magnetically isolated (EasySep Mouse CD4^+^CD25^+^ Regulatory T cell isolation kit II, StemCell) and 200,000 Gli2ΔN2 Tregs or 200,000 Gli2ΔC2 Tregs were injected i.p. into WT mice 2 days before and on day 7 of Oxa treatment (Supplemental Figure 5E). WT Oxa-treated control mice were injected with PBS. Mice were analyzed on day 14.

### Histological analysis of skin.

Ear pinnae sections were fixed in Bouin’s solution (MilliporeSigma). H&E staining was as described (76). Images were taken using a Zeiss Axioplan microscope (×10 magnification). For immunofluorescence, ear sections were frozen in OCT (Thermo Fisher Scientific) and cut into 7.5-μm sections. Nonspecific binding was blocked by incubation with 0.2% fish gelatin (MilliporeSigma) and 0.1% Tween (BioRad) in PBS (Life Technologies). Cells were stained with primary unconjugated anti-Shh (1:50 dilution; 171018; Santa Cruz Biotechnology), followed by incubation with donkey anti-goat IgG Alexa Fluor 594 (1:500 dilution; Invitrogen) secondary antibody in blocking buffer. Slides were mounted with Gold antifade reagent with DAPI (Invitrogen) and visualized using a Leica upright 3-color microscope (×10 magnification). Quantification of dermal/epidermal thickness and immunofluorescence image analysis were carried out using Fiji software.

### In vitro Treg cell suppression assay.

CD4^+^CD25^–^ T cells and CD4^+^CD25^+^ Treg cells from WT and Gli2ΔC2 spleens of Oxa-treated mice were FACS-sorted. WT CD4^+^ T cells were first stained with CFSE and then cocultured with Tregs at 1:1 and 1:4 ratios as described (77) for 4 days.

### Measurement of IgE and cytokines.

Serum was obtained and IgE levels measured by ELISA (eBioscience kit) as described (78) according to the manufacturer’s instructions. Supernatants were collected after 4 days of coculture of CD4^+^ T cells and Tregs. Active TGF-β levels were measured by ELISA (Invitrogen) according to the manufacturer’s instructions.

Ear pinnae were homogenized and centrifuged at 2330 *g* for 20 minutes to collect skin supernatants. Cytokines from homogenates were measured by multiplex immunoassays using Firefly from Abcam, according to the manufacturer’s protocol.

### Human samples.

Human peripheral blood mononuclear cells (PBMCs) were freshly isolated using Ficoll-Paque (MilliporeSigma) from randomly selected unknown leukocyte-cone donors from the United Kingdom NHS Blood and Transplant Centre. After magnetic separation (EasySep Isolation Kit, StemCell), naive CD4^+^ T cells (CD3^+^CD4^+^CD45RA^+^CD45RO^–^) were differentiated under iTreg conditions at 1 × 10^6^ cells/mL. Cells were stimulated with plate-bound anti-CD3 (5 μg/mL, UCHT1; eBioscience), soluble anti-CD28 (1 μg/mL, eBioscience), IL-2 (100 IU/mL, eBioscience) and TGF-β1 (5 ng/mL, Peprotech). Recombinant Shh (0.5 μg/mL, R&D Systems) was added, cells were incubated for 5 days and analyzed for intracellular FOXP3 expression.

### RNA extraction and quantitative RT-PCR (QRT-PCR) analysis.

RNA was extracted using the Arcturus PicoPure RNA isolation kit (Applied Biosystems), following the manufacturer’s instructions. RNA conversion to cDNA and QRT-PCR were as described (78), using primers purchased from Qiagen.

### RNA-sequencing.

After induction of AD by the Oxa protocol, skin cell suspensions from 2 groups of 3 mice each from WT, Gli2ΔN2, and Gli2ΔC2 mice were obtained. Cells were stained and live cells sorted (CD45^+^CD3^+^γδTCR^–^CD4^+^CD8^–^), RNA extracted, and RNA quality determined with a Bioanalyzer 2100 (Agilent Technologies). cDNA conversion and RNA-seq, performed by UCL Genomics on a NextSeq 500 system (Illumina), were as described (28). All RNAsequencing data were deposited in the NCBI’s Gene Expression Omnibus database (GEO GSE117338). The normalized estimates of transcript abundance expressed as RPKM (reads per kilobase of transcripts per million mapped reads) were generated using the Bioconductor package DESeq2. DEGs were determined using Ebayes analysis (*P* < 0.05) from the limma package in Bioconductor. CRAN package ade4 was used for PCA analysis.

### Statistics.

An unpaired 2-tailed Student’s *t* test was performed for comparison of the experimental groups, unless otherwise stated. For statistical analysis of multiple comparisons ANOVA was used where stated. For data shown in Figure 8, F and G (left), paired 2-tailed *t* test was used. *P* less than 0.05 was considered statistically significant.

### Study approval.

Mouse studies were carried out under UK Home Office regulations, following ethical review at University College London. Experiments with human cells were ethically approved by the area NHS Research Ethics Committee. Cells were obtained from leukocyte cones from anonymous donors to our national blood service, so informed consent to take part in this specific study was not required.

## Author contributions

EP and TC conceived and designed experiments and wrote the paper.EP, AS, CIL, DCY, SR, AV, IR, MMC, MO, and RFLO designed and performed experiments and analyzed data. CIL, DCY, SR, MO, and RFLO critically reviewed the manuscript.

## Supplementary Material

Supplemental data

## Version 1 07/02/2019

Electronic publication

## Version 2 08/01/2019

Print issue publication

## References

[1] Bieber T (2008). Mechanisms of disease: Atopic dermatitis. New Engl J Med.

[2] Gittler JK, Krueger JG, Guttman-Yassky E (2013). Atopic dermatitis results in intrinsic barrier and immune abnormalities: implications for contact dermatitis. J Allergy Clin Immunol.

[3] Koga C, Kabashima K, Shiraishi N, Kobayashi M, Tokura Y (2008). Possible pathogenic role of Th17 cells for atopic dermatitis. J Invest Dermatol.

[4] Bieber K (2017). Regulatory T cells suppress inflammation and blistering in pemphigoid diseases. Front Immunol.

[5] Dudda JC, Perdue N, Bachtanian E, Campbell DJ (2008). Foxp3+ regulatory T cells maintain immune homeostasis in the skin. J Exp Med.

[6] Kashiwagi M (2017). Direct control of regulatory I cells by keratinocytes. Nat Immunol.

[7] Malhotra N (2018). RORalpha-expressing T regulatory cells restrain allergic skin inflammation. Sci Immunol.

[8] Sanchez Rodriguez R (2014). Memory regulatory T cells reside in human skin. J Clin Invest.

[9] Ingham PW, Nakano Y, Seger C (2011). Mechanisms and functions of Hedgehog signalling across the metazoa. Nat Rev Genet.

[10] Briscoe J, Therond PP (2013). The mechanisms of Hedgehog signalling and its roles in development and disease. Nat Rev Mol Cell Bio.

[11] Ramsbottom SA, Pownall ME (2016). Regulation of Hedgehog signalling inside and outside the cell. J Dev Biol.

[12] Teglund S, Toftgard R (2010). Hedgehog beyond medulloblastoma and basal cell carcinoma. Biochim Biophys Acta.

[13] Takebe N (2015). Targeting Notch, Hedgehog, and Wnt pathways in cancer stem cells: clinical update. Nat Rev Clin Oncol.

[14] Hager-Theodorides AL, Dessens JT, Outram SV, Crompton T (2005). The transcription factor Gli3 regulates differentiation of fetal CD4(–)CD8(–) double-negative thymocytes. Blood.

[15] Hager-Theodorides AL, Furmanski AL, Ross SE, Outram SV, Rowbotham NJ, Crompton T (2009). The Gli3 transcription factor expressed in the thymus stroma controls thymocyte negative selection via Hedgehog-dependent and -independent mechanisms. J Immunol.

[16] Outram SV, Varas A, Pepicelli CV, Crompton T (2000). Hedgehog signaling regulates differentiation from double-negative to double-positive thymocyte. Immunity.

[17] Rowbotham NJ (2007). Activation of the Hedgehog signaling pathway in T-lineage cells inhibits TCR repertoire selection in the thymus and peripheral T-cell activation. Blood.

[18] Rowbotham NJ (2008). Repression of hedgehog signal transduction in T-lineage cells increases TCR-induced activation and proliferation. Cell Cycle.

[19] Shah DK, Hager-Theodorides AL, Outram SV, Ross SE, Varas A, Crompton T (2004). Reduced thymocyte development in sonic hedgehog knockout embryos. J Immunol.

[20] Solanki A (2018). Gli3 in fetal thymic epithelial cells promotes thymocyte positive selection and differentiation by repression of Shh. Development.

[21] Outram SV (2009). Indian hedgehog (Ihh) both promotes and restricts thymocyte differentiation. Blood.

[22] de la Roche M (2013). Hedgehog signaling controls T cell killing at the immunological synapse. Science.

[23] Furmanski AL (2013). Tissue-derived hedgehog proteins modulate Th differentiation and disease. J Immunol.

[24] Furmanski AL (2015). The transcriptional activator Gli2 modulates T-cell receptor signalling through attenuation of AP-1 and NFkappaB activity. J Cell Sci.

[25] Standing ASI, Yanez DC, Ross R, Crompton T, Furmanski AL (2017). Frontline science: Shh production and Gli signaling is activated in vivo in lung, enhancing the Th2 response during a murine model of allergic asthma. J Leukoc Biol.

[26] Man MQ (2008). Characterization of a hapten-induced, murine model with multiple features of atopic dermatitis: structural, immunologic, and biochemical changes following single versus multiple oxazolone challenges. J Invest Dermatol.

[27] Balaskas N (2012). Gene regulatory logic for reading the Sonic Hedgehog signaling gradient in the vertebrate neural tube. Cell.

[28] Solanki A, Lau CI, Saldana JI, Ross S, Crompton T (2017). The transcription factor Gli3 promotes B cell development in fetal liver through repression of Shh. J Exp Med.

[29] Munchhof MJ (2012). Discovery of PF-04449913, a potent and orally bioavailable inhibitor of smoothened. ACS Med Chem Lett.

[30] Wagner AJ (2015). A phase I study of PF-04449913, an oral hedgehog inhibitor, in patients with advanced solid tumors. Clin Cancer Res.

[31] Son ED (2014). Staphylococcus aureus inhibits terminal differentiation of normal human keratinocytes by stimulating interleukin-6 secretion. J Dermatol Sci.

[32] Nakamura Y, Franchi L, Kambe N, Meng G, Strober W, Nunez G (2012). Critical role for mast cells in interleukin-1beta-driven skin inflammation associated with an activating mutation in the nlrp3 protein. Immunity.

[33] Czarnowicki T, Santamaria-Babi LF, Guttman-Yassky E (2017). Circulating CLA(+) T cells in atopic dermatitis and their possible role as peripheral biomarkers. Allergy.

[34] Lu Y, Li J, Cheng J, Lubahn DB (2015). Genes targeted by the Hedgehog-signaling pathway can be regulated by estrogen related receptor beta. BMC Mol Biol.

[35] Lau CI, Barbarulo A, Solanki A, Saldana JI, Crompton T (2017). The kinesin motor protein Kif7 is required for T-cell development and normal MHC expression on thymic epithelial cells (TEC) in the thymus. Oncotarget.

[36] Min Y (2016). Cereblon negatively regulates TLR4 signaling through the attenuation of ubiquitination of TRAF6. Cell Death Dis.

[37] Wang JL, Qi Z, Li YH, Zhao HM, Chen YG, Fu W (2017). TGFbeta induced factor homeobox 1 promotes colorectal cancer development through activating Wnt/beta-catenin signaling. Oncotarget.

[38] Holla S (2016). Mycobacteria-responsive sonic hedgehog signaling mediates programmed death-ligand 1- and prostaglandin E2-induced regulatory T cell expansion. Sci Rep.

[39] El Andaloussi A, Graves S, Meng F, Mandal M, Mashayekhi M, Aifantis I (2006). Hedgehog signaling controls thymocyte progenitor homeostasis and differentiation in the thymus. Nat Immunol.

[40] Saldana JI (2016). Sonic Hedgehog regulates thymic epithelial cell differentiation. J Autoimmun.

[41] Woo WM, Zhen HH, Oro AE (2012). Shh maintains dermal papilla identity and hair morphogenesis via a Noggin-Shh regulatory loop. Genes Dev.

[42] Konkel JE (2017). Transforming growth factor-beta signaling in regulatory T cells controls T helper-17 cells and tissue-specific immune responses. Immunity.

[43] Tran DQ (2012). TGF-beta: the sword, the wand, and the shield of FOXP3(+) regulatory T cells. J Mol Cell Biol.

[44] Chen ML, Yan BS, Bando Y, Kuchroo VK, Weiner HL (2008). Latency-associated peptide identifies a novel CD4+CD25+ regulatory T cell subset with TGFbeta-mediated function and enhanced suppression of experimental autoimmune encephalomyelitis. J Immunol.

[45] Worthington JJ, Klementowicz JE, Travis MA (2011). TGFbeta: a sleeping giant awoken by integrins. Trends Biochem Sci.

[46] Fahlen L (2005). T cells that cannot respond to TGF-beta escape control by CD4(+)CD25(+) regulatory T cells. J Exp Med.

[47] Liu H, Hu B, Xu D, Liew FY (2003). CD4+CD25+ regulatory T cells cure murine colitis: the role of IL-10, TGF-beta, and CTLA4. J Immunol.

[48] Zhang X, Reddy J, Ochi H, Frenkel D, Kuchroo VK, Weiner HL (2006). Recovery from experimental allergic encephalomyelitis is TGF-beta dependent and associated with increases in CD4+LAP+ and CD4+CD25+ T cells. Int Immunol.

[49] Suarez-Farinas M (2011). Nonlesional atopic dermatitis skin is characterized by broad terminal differentiation defects and variable immune abnormalities. J Allergy Clin Immunol.

[50] Eggenschwiler JT, Espinoza E, Anderson KV (2001). Rab23 is an essential negative regulator of the mouse Sonic hedgehog signalling pathway. Nature.

[51] Itou J, Taniguchi N, Oishi I, Kawakami H, Lotz M, Kawakami Y (2011). HMGB factors are required for posterior digit development through integrating signaling pathway activities. Dev Dyn.

[52] Pazin DE, Albrecht KH (2009). Developmental expression of Smoc1 and Smoc2 suggests potential roles in fetal gonad and reproductive tract differentiation. Dev Dyn.

[53] Tiberi L (2014). A BCL6/BCOR/SIRT1 complex triggers neurogenesis and suppresses medulloblastoma by repressing Sonic Hedgehog signaling. Cancer Cell.

[54] Chen H, Wang J, Yang H, Chen D, Li P (2016). Association between FOXM1 and hedgehog signaling pathway in human cervical carcinoma by tissue microarray analysis. Oncol Lett.

[55] Zheng X (2013). Role of the Hedgehog pathway in hepatocellular carcinoma (review). Oncol Rep.

[56] Paternoster L (2011). Meta-analysis of genome-wide association studies identifies three new risk loci for atopic dermatitis. Nat Genet.

[57] Hirota T (2012). Genome-wide association study identifies eight new susceptibility loci for atopic dermatitis in the Japanese population. Nat Genet.

[58] Verhagen J (2006). Absence of T-regulatory cell expression and function in atopic dermatitis skin. J Allergy Clin Immunol.

[59] Blaydon DC (2011). Inflammatory skin and bowel disease linked to ADAM17 deletion. N Engl J Med.

[60] Murthy A, Shao YW, Narala SR, Molyneux SD, Zuniga-Pflucker JC, Khokha R (2012). Notch activation by the metalloproteinase ADAM17 regulates myeloproliferation and atopic barrier immunity by suppressing epithelial cytokine synthesis. Immunity.

[61] Gudjonsson JE (2010). Evidence for altered Wnt signaling in psoriatic skin. J Invest Dermatol.

[62] Perez-Moreno M, Davis MA, Wong E, Pasolli HA, Reynolds AB, Fuchs E (2006). p120-catenin mediates inflammatory responses in the skin. Cell.

[63] Wang W, Yu X, Wu C, Jin H (2017). IL-36gamma inhibits differentiation and induces inflammation of keratinocyte via Wnt signaling pathway in psoriasis. Int J Med Sci.

[64] Alvarez JI (2011). The Hedgehog pathway promotes blood-brain barrier integrity and CNS immune quiescence. Science.

[65] Caradu C, Guy A, James C, Reynaud A, Gadeau AP, Renault MA (2018). Endogenous Sonic Hedgehog limits inflammation and angiogenesis in the ischaemic skeletal muscle of mice. Cardiovasc Res.

[66] Singh VB, Singh MV, Gorantla S, Poluektova LY, Maggirwar SB (2016). Smoothened agonist reduces human immunodeficiency virus type-1-induced blood-brain barrier breakdown in humanized mice. Sci Rep.

[67] Singh VB, Singh MV, Piekna-Przybylska D, Gorantla S, Poluektova LY, Maggirwar SB (2017). Sonic Hedgehog mimetic prevents leukocyte infiltration into the CNS during acute HIV infection. Sci Rep.

[68] van Dop WA (2010). Loss of Indian Hedgehog activates multiple aspects of a wound healing response in the mouse intestine. Gastroenterology.

[69] Zacharias WJ (2010). Hedgehog is an anti-inflammatory epithelial signal for the intestinal lamina propria. Gastroenterology.

[70] Zhou XY, Liu ZQ, Jang F, Xiang CN, Li Y, He YZ (2012). Autocrine Sonic Hedgehog attenuates inflammation in cerulein-induced acute pancreatitis in mice via upregulation of IL-10. PLoS One.

[71] Otsuka A (2015). Hedgehog pathway inhibitors promote adaptive immune responses in basal cell carcinoma. Clin Cancer Res.

[72] Islam SA (2011). Mouse CCL8, a CCR8 agonist, promotes atopic dermatitis by recruiting IL-5(+) T(H)2 cells. Nat Immunol.

[73] Gandhi NA, Bennett BL, Graham NM, Pirozzi G, Stahl N, Yancopoulos GD (2016). Targeting key proximal drivers of type 2 inflammation in disease. Nat Rev Drug Discov.

[74] Chiang C (1996). Cyclopia and defective axial patterning in mice lacking Sonic hedgehog gene function. Nature.

[75] Jin H, He R, Oyoshi M, Geha RS (2009). Animal models of atopic dermatitis. J Invest Dermatol.

[76] Lau CI, Outram SV, Saldana JI, Furmanski AL, Dessens JT, Crompton T (2012). Regulation of murine normal and stress-induced erythropoiesis by Desert Hedgehog. Blood.

[77] Lau CI, Yanez DC, Solanki A, Papaioannou E, Saldana JI, Crompton T (2018). Foxa1 and Foxa2 in thymic epithelial cells (TEC) regulate medullary TEC and regulatory T-cell maturation. J Autoimmun.

[78] Yanez DC (2019). IFITM proteins drive type 2 T helper cell differentiation and exacerbate allergic airway inflammation. Eur J Immunol.

