# Supplementary material for: Sonic Hedgehog signaling limits atopic dermatitis via Gli2-driven immune regulation
Source: J Clin Invest. 2019 Jul 2;129(8):3153–70. doi: 10.1172/JCI125170 (PMC6668675; doi:10.1172/JCI125170)
Supplement: Supplemental data [file jci-129-125170-s134.pdf]

Legends for Supplementary Figures and Tables

Figure S1: Oxa-induced model of chronic atopic dermatitis.

Data from two independent experiments with 3 mice per group unless otherwise stated for untreated (black) and Oxa-treated (red); each symbol represents an individual animal; measurements taken at termination of the experimental protocol shown in Figure 1A (day 14).

A. Representative images of ear pinnae from WT Oxa-treated (left) and WT control (right).
B. Representative H&E images of ear sections from untreated and Oxa-treated WT mice on termination of the experimental protocol shown in Figure 1A (day 14). Scale bar: 100μm. Plots: dermal and epidermal thickness of control and Oxa-treated WT.
C. Percentage of skin T-cells that express IFN-γ, IL-13 and IL-17 in control and Oxa-treated WT mice measured by intracellular cytokine staining and flow cytometry.
D. Serum IgE concentration from WT control and Oxa-treated WT mice, measured by ELISA.
E. Numbers per ear of skin $CD4^+$ and $CD8^+$ T-cells and T_{regs} ($CD4^+CD25^+icFoxp3^+$) isolated from control and Oxa-treated mice measured by flow cytometry.
F. *Filaggrin*, *Ifng*, and *Il4* expression (QRT-PCR) in ear homogenates from control untreated WT and Rag1-KO; and Oxa-treated WT and Rag1-KO. Data were generated from two independent experiments with 3-6 mice per group and a 2-way ANOVA statistical analysis was performed.

Statistics: 2-tailed unpaired student's t-test unless otherwise stated. Plots: mean±SEM. *$p<0.05$, **$p<0.01$, ***$p<0.001$ and ****$p<0.0001$.

Figure S2: Smo inhibition exacerbates skin inflammation upon induction of atopic dermatitis.

Data from 7 mice per group (unless otherwise stated) for WT control (without Smo-inh, DMSO only, black) and Smo-inh-injected (red) mice; each symbol represents an individual animal.

A. Representative H&E images in absence of Oxa-treatment for skin from control (DMSO-only injected) and Smo-inh injected WT mice. Mice were injected with Smo-inhibitor daily for 14 days. Plots: percentage and number of skin leukocytes ($CD45^+$) and $CD4^+$ and $CD8^+$ T-cells from mice in the absence of Oxa-treatment from control and Smo-inh injected WT mice, measured by flow cytometry.

B. Percentage and number in Oxa-treated mice of skin $CD4^+$ and $CD8^+$T-cells from control or Smo-inh injected groups.

C. Percentage of skin $CD4^+$ and $CD8^+$ T-cells (gated on $CD45^+CD3^+\gamma\delta$TCR) (upper plots) and number of $CD4^+$ and $CD8^+$ T-cells isolated from ears (lower plots), from Oxa-treated WT mice from control (without Smo-inh,) and Smo-inh injected groups.

D. Percentage of skin $CD4^+$ T-cells that express IL4 and IL13 from Oxa-treated WT mice from control and Smo-inh injected groups, measured by intracellular cytokine staining.

Statistics: 2-tailed unpaired student's t-test. Plots:mean±SEM.

Figure S3: T cell populations in Gli2ΔC2 and WT mice on induction of atopic dermatitis.

Data from two independent experiments with at least 6 Oxa-treated mice per group. Black circles represent WT mice while red squares represent Gli2ΔC2 mice. Representative FACS plots of CD4 and CD8 staining of dLNs from Oxa-treated WT (left) and Gli2ΔC2 (right) mice. Plots: percentage of $CD4^+$ and $CD8^+$ T-cells in dLNs.

A. Percentage of $CD69^+$ cells gated on $CD4^+$ (left) or $CD8^+$ (right) from dLNs of Oxa-treated WT and Gli2ΔC2 mice.

Statistics: 2-tailed unpaired student's t-test. Plots: each symbol represents an individual mouse and plots show mean±SEM *p<0.05, **p<0.01 and ***p<0.001.

Figure S4: T-cell populations in Gli2ΔN2 and WT mice on induction of atopic dermatitis.

Data were generated from two independent experiments with at least 6 Oxa-treated mice per group. Black circles: WT; red squares: Gli2ΔN2; each symbol represents an individual mouse and plots show mean±SEM.

A. Representative FACS plots of dLNs from WT (left) and Gli2ΔN2 (right) Oxa-treated mice. Plots: percentage of $CD4^+$ and $CD8^+$ T-cells in dLNs of WT and Gli2ΔN2 mice.
B. Representative FACS plots showing CD62L and CD44 staining from dLN from WT (left) and Gli2ΔN2 (right) Oxa-treated mice, giving the percentage of cells in the regions shown. Plots: percentages of naïve ($CD62L^+CD44^-$), T_{cm} ($CD62L^-CD44^+$) and T_{effm} ($CD62L^+CD44^+$) $CD4^+$ and $CD8^+$ T-cells from dLNs of WT and Gli2ΔN2 treated mice.

Statistics: 2-tailed unpaired student's t-test. **p<0.01 and ****p<0.0001.

Figure S5: Inhibition of Gli2-mediated transcription compromises immune regulation

A-B, F: Plots: each symbol represents an individual animal for Gli2ΔN2 (black), WT (red) and Gli2ΔC2 (blue), showing mean±SEM.

A. Percentage of T_{reg} ($CD4^{+}CD25^{+}Foxp3^{+}$) in dLNs from Oxa-treated Gli2ΔN2 WT and Gli2ΔC2.

B. Percentage of $Klrg1^{+}$ cells in the $CD4^{+}CD25^{+}Foxp3^{+}$ T_{reg} population from dLNs of Oxa-treated Gli2ΔN2, WT and Gli2ΔC2 groups. Histograms:representative anti-Klrg staining from dLNs for the three experimental groups gated on $CD4^{+}CD25^{+}Foxp3^{+}$ T_{reg} population.

C. Dot plots: gating strategy and percentage of cells in regions shown, for analysis of T_{reg} populations ($CD4^{+}CD25^{+}icFoxp3^{+}$) from Oxa-treated WT, Gli2ΔN2 and Gli2ΔC2 dLNs. Histograms: icCTLA-4, cell surface CD44 and icKi67 staining gated on $CD4^{+}CD25^{+}Foxp3^{+}$ T_{reg} cells. Percentage of cells in marker or region is given.

D. Time-line of antibody injections (anti-CD25 or control igG) and Oxa-treatment for experiment shown in Figure 7A-F.

E. Time-line of adoptive transfer of T_{regs} and Oxa-application for experiment shown in Figure 7G-L.

F. Adoptive transfer experiments into Oxa-treated WT mice: purified $CD4^{+}CD25^{+}$ (T_{reg}) from spleens of Oxa-treated Gli2ΔN2 (Gli2ΔN2-adoptive-transfer group, black) or Gli2ΔC2 mice (Gli2ΔC2-adoptive-transfer group, blue) were injected into WT mice as shown in Figure S5E, and compared to Oxa-treated WT control mice (control group; labelled WT, red). Plots:

percentage of cells positive with ic cytokine staining, gating on $CD4^+$ T-cells, from skin from the three groups of mice at the end of Oxa-treatment (day 14).

Statistics: A, B, F: one-way ANOVA; *p<0.05 and **p<0.01.

Supplementary Tables

Table S1: Gene list of intersection between 3000 genes that contributed most to PC2 and 1500 most DEG between RNA-seq datasets from Oxa-treated WT and Gli2ΔC2 skin CD4 T-cells.

Table S2: Gene list of intersection between 3000 genes that contributed most to PC2 and 2500 most DEG between RNA-seq datasets from Oxa-treated WT and Gli2ΔN2 skin CD4 T-cells.

Table S3: Expression values of *Shh* and *Gli3* in RNA-seq datasets from Oxa-treated WT, Gli2ΔC2 and Gli2ΔN2 skin CD4 T-cells.

Table S4: List of antibodies used in flow cytometry experiments.

Figure S1

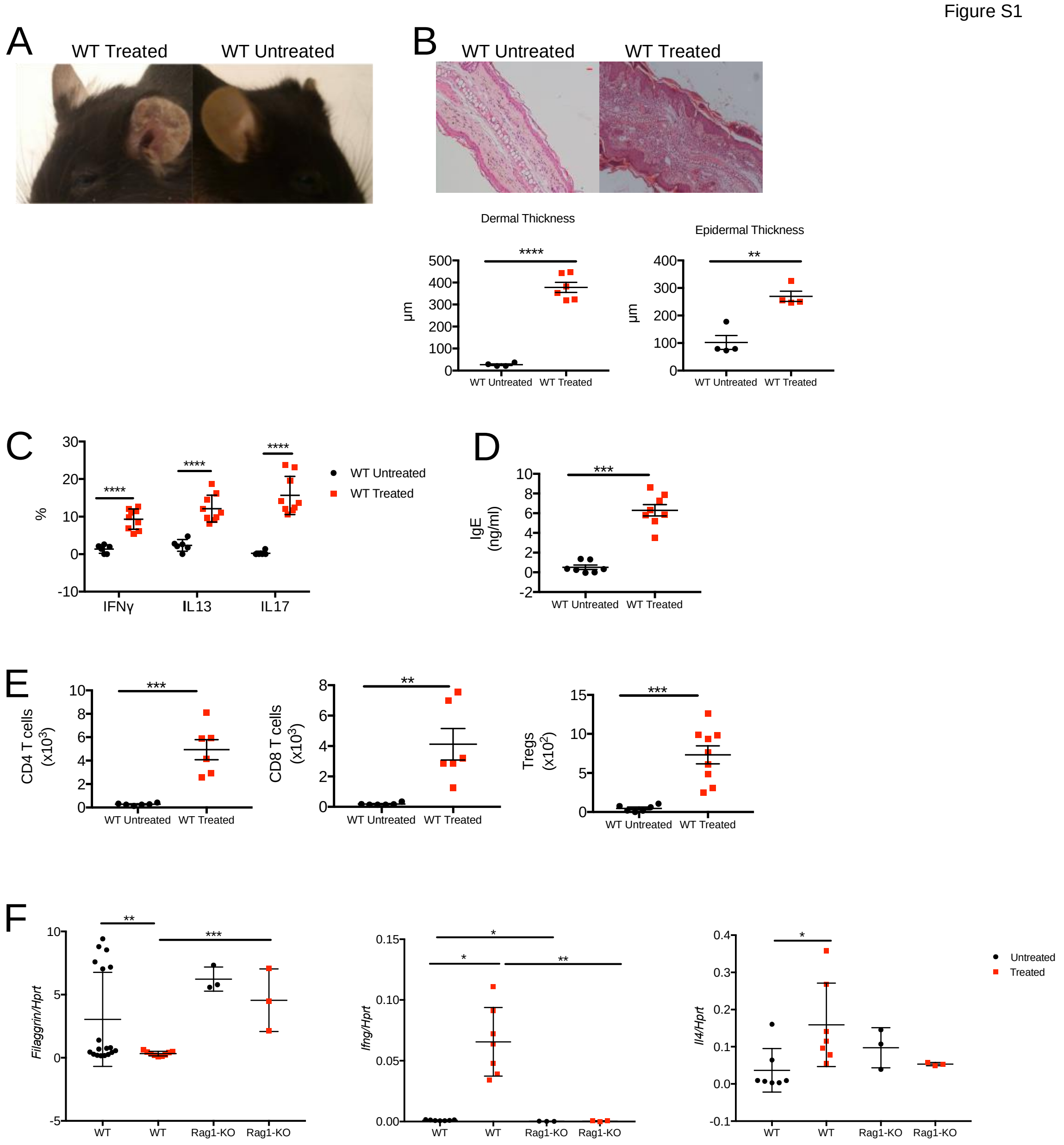
A
WT Treated
WT Untreated
B
WT Untreated
WT Treated
Dermal Thickness
Epidermal Thickness
μm
WT Untreated WT Treated
C
%
IFNγ
IL13
IL17
WT Untreated
WT Treated
D
IgE (ng/ml)
E
CD4 T cells ($x10^3$)
CD8 T cells ($x10^3$)
Tregs ($x10^2$)
F
Filaggrin/Hprt
Ifng/Hprt
Il4/Hprt
WT
Rag1-KO
Untreated
Treated

Figure S2

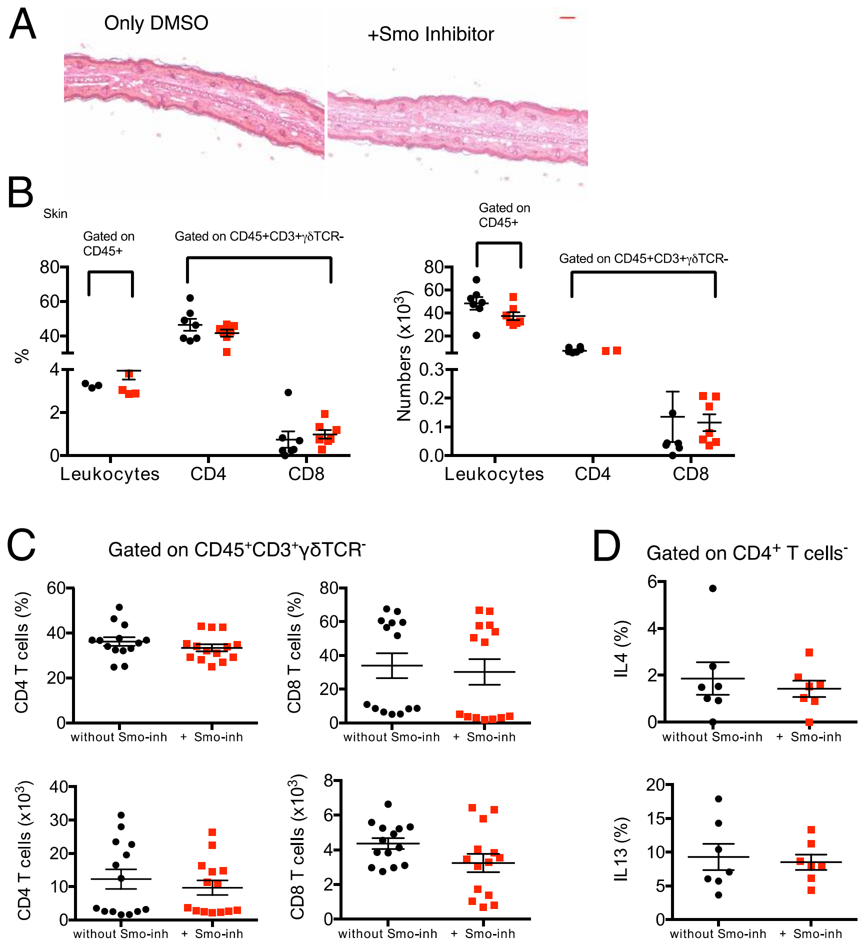
A
Only DMSO
+Smo Inhibitor
B
Skin
Gated on CD45+
Gated on CD45+CD3+γδTCR-
%
Leukocytes
CD4
CD8
Numbers (x10³)
C
Gated on CD45⁺CD3⁺γδTCR⁻
CD4 T cells (%)
CD8 T cells (%)
CD4 T cells (x10³)
CD8 T cells (x10³)
without Smo-inh
+ Smo-inh
D
Gated on CD4⁺ T cells⁻
IL4 (%)
IL 13 (%)

Figure S3

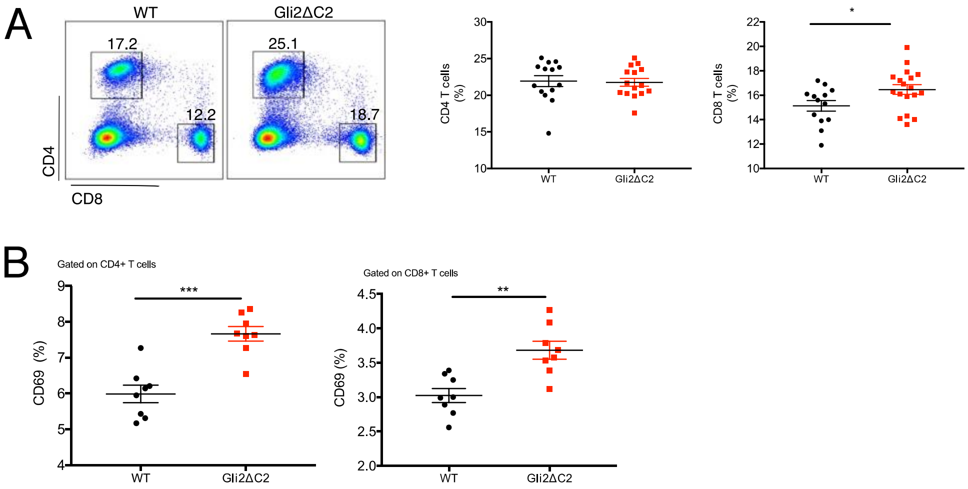

A
WT
Gli2ΔC2
17.2
12.2
25.1
18.7
CD4
CD8
CD4 T cells (%)
CD8 T cells (%)
*
B
Gated on CD4+ T cells
Gated on CD8+ T cells
***
**
CD69 (%)
WT
Gli2ΔC2

Figure S4

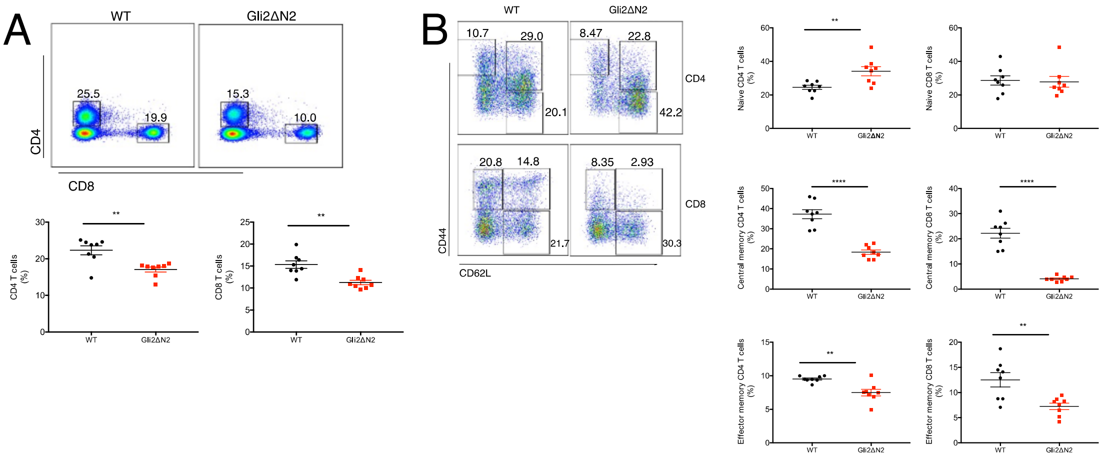

A
WT
Gli2ΔN2
25.5
19.9
15.3
10.0
CD4
CD8
CD4 T cells (%)
CD8 T cells (%)
B
10.7
29.0
20.1
8.47
22.8
42.2
20.8
14.8
21.7
8.35
2.93
30.3
CD44
CD62L
Naive CD4 T cells (%)
Naive CD8 T cells (%)
Central memory CD4 T cells (%)
Central memory CD8 T cells (%)
Effector memory CD4 T cells (%)
Effector memory CD8 T cells (%)

Figure S5

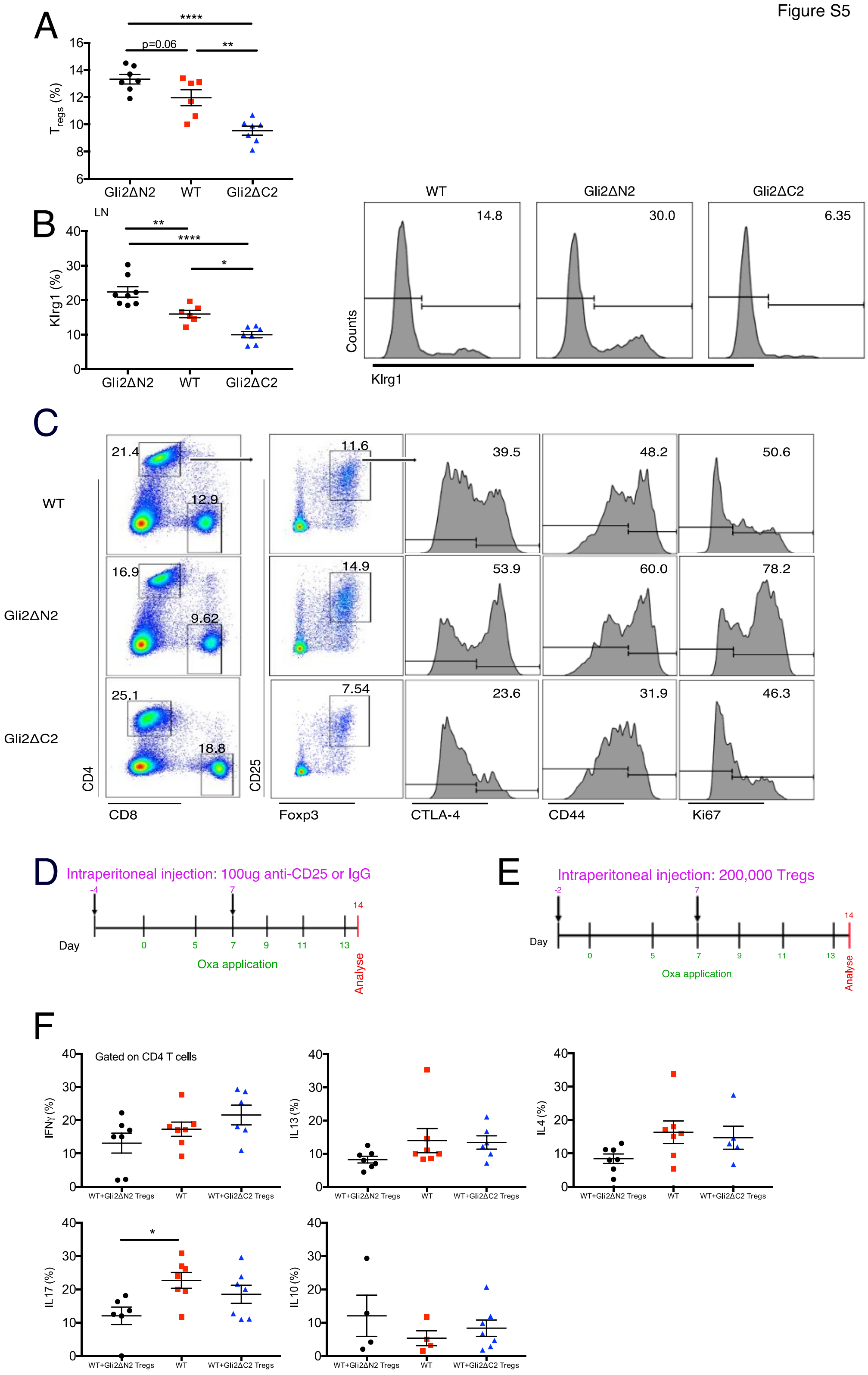

A
****
p=0.06
**
Tregs (%)
Gli2ΔN2
WT
Gli2ΔC2
B
LN
**
****
*
Klrg1 (%)
WT
Gli2ΔN2
Gli2ΔC2
14.8
30.0
6.35
Counts
Klrg1
C
WT
Gli2ΔN2
Gli2ΔC2
21.4
12.9
11.6
39.5
48.2
50.6
16.9
9.62
14.9
53.9
60.0
78.2
25.1
18.8
7.54
23.6
31.9
46.3
CD4
CD8
CD25
Foxp3
CTLA-4
CD44
Ki67
D
Intraperitoneal injection: 100ug anti-CD25 or IgG
Day
Oxa application
Analyse
E
Intraperitoneal injection: 200,000 Tregs
Day
Oxa application
Analyse
F
Gated on CD4 T cells
IFNγ (%)
IL13 (%)
IL4 (%)
IL17 (%)
IL10 (%)
WT+Gli2ΔN2 Tregs
WT
WT+Gli2ΔC2 Tregs
*

Table S1 (Relating to Figure 6): Gene list of intersection between 3000 genes that contributed most to PC2 and 1500 most DEG between RNAseq datasets from Oxa-treated WT and Gli2C∆2 skin CD4 T cells

Tspyl4	Tbk1	Flrt3	Gtf2a2	Ssu72	Dlgap3
Rbks	Vamp8	Slu7	Gng5	Gnpnat1	Dynlrb1
N4bp2l1	Cfp	Cxcl16	Scarb2	Bcl2a1a	C1qc
Ctsb	Anpep	Snx14	Eapp	Ubb	Itfg1
Cd3d	Grb2	Rbm18	Tspo	Snx6	Ostf1
Slc27a4	Ciita	Sdf4	Clec4n	Nrg1	Mgl2
Tspyl1	Cox7b	Ormdl2	Deb1	Pcgf5	Hist1h2bn
Abhd17b	Cstb	Sh3bgr	Mmp13	Kctd1	Polr2c
Adssl1	Samsn1	Commd9	B2m	C1qb	Card9
Ctss	Tgfbi	Cox4i1	Ctsz	Pdxp	Ypel5
Saraf	Pafah1b3	AI837181	C1qa	Cd86	Clec7a
Fcgr2b	Siglech	Snx17	Ptpn2	P2rx4	Mrc1
Brdt	Il4i1	Tdrd7	Fam221a	Serpinb1a	Mzb1
Cnot8	Apool	Klk1	Npc2	Pi4kb	Cd68
Zfp677	Chmp1a	Srp9	Cd74	AF251705	Ccr9
Asb8	Msr1	Ms4a7	Klre1	Mitd1	Zmat5
Dyrk4	Erbb3	Dirc2	Vimp	Galm	Esd
Paip2	Nos2	AI467606	Pira6	Iigp1	Syp
Ndufv2	Cep57l1	Morc3	Clec4d	Gcm2	Klra3
Emc4	AW112010	Aoah	Efhb	Atp6v1f	Lrrc8c
Timd4	Amz1	Kcnj16	Pdhb	Jmjd6	Mtfr1l
St7l	Spib	Efr3b	Sdcbp	Pcdhga7	Sirpa
Mfsd1	Tlr12	Rfwd2	Tarm1	Zfr	Insig1
Alkbh7	Smim5	Map1lc3b	Mkln1	Atp5c1	Vamp3
Cib1	Tmem63a	Tram1	Ippk	Ak4	Il12rb2
Them4	Vwa5a	Dnah7a	Vti1b	G6pdx	Apeh
Slc22a4	Sqstm1	Aox2	AU019990	Dthd1	Ifltd1
Rgs9bp	Phex	Gm15694	Vdac2	Fundc1	Il1a
Rer1	Kcnn4	Rps6ka4	Iqcg	Gk5	Cd3g
Il1rn	Gm4262	Mtch2	Ilk	Acot13	Adap2
Tmem86a	Pnp	Eno1	Plaur	Ermap	Polr3a
Ndufv3	Eepd1	Muc20	Nfe2	C3ar1	Ly6a
Map6d1	Mpeg1	Hsd11b1	Alg14	Cebpa	Pcp2
Blnk	Tmem43	Pigu	Pknox1	Fam173b	Dnah2
Aip	Wscd1	Prss2	Sqle	Arfrp1	Abt1
Eif1	Ctsh	Ctsd	Smad2	Yipf3	Dhrs9
Slfn5	Ccl24	Hes5	Stx8	Psma1	Syngr2
Fhl2	Zfp148	Tor3a	Spry1	Treml4	Rhot1
Sarnp	Arg1	P2rx7	Pja2	Jtb	Fcrlb
Ginm1	Etv5	Necap2	Ccdc71	Snord22	Ncf2
Cd79b	Klf2	Fcer1g	Snord89	Ctrc	Pdcd10
Tmx3	Tyrobp	Scimp	Fcgr3	Fdft1	Sh2d3c
Srgn	Npy	Slc25a46	Ggta1	Klrc1	Lnpep
Epb4.1l5	Ddx58	Itgb3bp	Krtcap2	Mob2	Gimap6
Fdps	Hddc3	Actr10	Cd53	Actr1a	Pgf
Gm5150	Med21	Uqcrb	Lamtor2	Aig1	Batf
Mvb12a	Arhgap17	Qars	Ifit1bl1	Snord4a	Cd48
Atg14	Wdr11	Mmp19	Cluap1	B430306N03Rik	Mir3068
Gm1045	Susd3	Wdr47	Zbp1	Lsm1	Gbp5
Acp6	Gm1821	Tnfrsf9	Mob3b	Rtp4	Psmb1
Mrps6	Gm14207	Pdk1	Ugp2	Nsmce1	Irg1
Gngt2	Zfp949	Bard1	Gpsm1	Fam195b	Gm996
Myl6	Snap23	Asah1	Mdh1	Stx3	Tmbim4
Chmp2a	Tmem9b	Zfp458	Irf1	Gabarap	Tsnax
Dynlt1f	Slc25a17	Lrp12	Sgpl1	AA986860	Ccdc66

Entpd3	Lss	Nit2	Rmnd1	Hsdl2	Ppil2
Vegfa	Errfi1	Arpc3	Zfp383	Flt3	Mynn
Ssr1	Lsp1	Spg21	Psenen	Phf11c	Sag
Nceh1	Ptcd2	Pomt2	Nub1	Wdr20	Pot1a
Bphl	Stard10	Crbn	Gm13830	Nxpe4	Rps6ka2
Ndufa1	Hectd2	Arpc2	Tgtp1	Gm5148	Tfcp2
Gimap9	Arl10	Gpr137b	Fdx1l	Gm10872	Sec22b
Usp40	Selk	Got1	AW146154	Lgals9	Csf2
Msra	Mcts2	Msrb1	Bud31	Cpne1	Ninj2
Ssr4	Lgals3bp	Fxyd5	Chmp5	Chp1	Cd6
Tmem234	Pex2	Ralgps2	Ccl20	Cmpk2	Rab7
Gosr2	Copg2	Lilrb4a	B230217O12Rik	Panx1	Phf11a
Tnfrsf13c	Tmem39a	Slc14a1	Tmem8	Hagh	Dapl1
Ift88	Myl12b	Irf9	Nudt22	Zfp512	Ms4a4b
Trpm4	Ubl3	Tifa	Thap2	Tmem258	Wdr61
Slc41a3	Pld3	Cd37	Cyp51	Ndufb3	Mcph1
Mrps11	Cenpw	E2f8	Ncl	Fam168b	Sema4d
Lig1	Wbp11	B4galnt4	Brca1	Rab11fip3	Rqcd1
Cnot4	Gdpd3	Lasp1	Pim3	Depdc1a	Kctd17
Ctdp1	Nupr1l	Hus1	Tmem265	Jagn1	Crebbp
Tet2	Sec14l2	Dlgap5	Cyp4f13	Polr1b	Lnx2
Ovol2	Micall1	Rfx1	Surf2	Ssc4d	Mospd3
Gpr162	Lrrc61	Impad1	Ipo13	Purb	Man2a2
Gemin4	Tk1	Wfikkn2	Rrm1	Heatr1	Incenp
Ablim1	Pwp1	Hyal2	Tamm41	Ccnb2	Cic
Map3k2	Racgap1	Mocs1	Brd1	Ubr4	Srrm1
Med14	Zxdc	Slc12a2	Pold2	Slc29a1	Pdss2
Nucks1	Srsf9	Pfas	Krtcap3	Ube2c	Ccdc6
Ube2h	Il1rl1	Ncapd3	Prkch	Trp53bp2	Hist1h1b
Unc5a	Rad51ap1	Tst	Ranbp1	Tsc22d4	Pggt1b
Gpatch4	Pa2g4	Thoc1	Rassf7	Rasl11a	Ywhag
Hnrnpa3	Patz1	Tmem194b	Xpo4	Mybl2	Rfc1
Set	Irf6	Prune2	H2afx	Nup93	Myc
Slc7a7	Mrps28	Ksr1	Gak	Ppp1r3f	Ssbp3
Exd2	Nat10	Ubap2	Rdx	Pkdcc	Tet3
Itpr2	Acadl	Snora65	Smo	Baz1a	Pnn
Fmr1	Nol6	Paxip1	Xxylt1	Shmt1	Ddhd1
Pou2f1	Smarcal1	Naa40	Nrf1	Ehbp1l1	Aurkb
Ppid	Skp2	Klhdc4	Tmpo	Aasdh	Ung
Csnk2a1	Arhgap12	Kif20b	Mtfr2	Gcnt1	Adcy3
E2f7	Ntpcr	Sec14l1	Mmgt1	Uhrf1bp1	Fam53b
Zfp408	Zfp36l2	Qdpr	Ltb4r1	Nploc4	Usp48
Ssh1	Ipo7	Gata3	Fbxw7	Camsap2	Cenpf
Npm3	Shcbp1	Vti1a	Ttll10	Atmin	Asxl1
Cacnb1	Hspa2	Tgfbrap1	Kif13b	Syce1l	Zfp276
Msantd1	Dbil5	Zdhhc7	Hs6st1	Kmt2b	Plcxd1
Mirlet7i	Mettl1	Acad11	Nup188	Ruvbl1	Tnp2
Pprc1	Tardbp	Gm16157	Gm4532	Zfp598	Usp32
Alg6	C430002N11Rik	Msc	Depdc5	Ncoa5	Uhmk1
Pced1a	Gins2	Myo1h	Unc13d	Gcc2	Coprs
Marf1	Ube2t	Mrpl45	Txlna	Moap1	Cep170b
Ubr5	Syne4	Tomm40	Coro2b	Mir6902	Mir19a
Rmi2	Elf4	Slfn5os	Lrriq3	B230319C09Rik	C330027C09Rik

A930007I19Rik	Midn	Gbf1	Evpl	Ncapg	Dclk2
Traip	Firre	Ttyh3	Tab1	Memo1	Pip4k2c
Tfpi	Gabrr1	Gm15800	E130307A14Rik	Zbtb42	Amn1
Arhgef17	Ankdd1b	Phf7	Nbeal2	Gar1	Gsta3
Ndc1	Ska1	Tcf20	Arhgef7	Stk10	A630089N07Rik
Cenpe	Rdh1	Cryz	Alkbh4	Cox7a1	Lmtk2
Stmn1	Eomes	Nos1	Syt12	Dus2	Inf2
Myo6	Sntg2	Cntrob	Eif3a	Nin	Larp1
Arfgef2	Vrk1	Mpi	Ckap2	E2f1	Hmga2
Slc7a4	Gm6277	Mapk1ip1l	Wdr24	Tmem39b	Zc3h4
Snora3	Kif7	Ccdc34	Dnah11	Muc4	Sass6
Recql	Pom121	Wbp1l	Gpatch8	Gcnt4	Plekhs1
Cldn7	Elmo3	Pfdn2	Plk4	Gse1	Scrn3
Chd1	Ppm1f	Tagln3	Myb	Ubr2	Nudc
Rab3a	Zfp91	Kif18b	Dgkh	Plekhg3	Tbc1d32
Srsf10	Olfr530	Fadd	Gcfc2	Ylpm1	Mir6385
Abhd1	Syvn1	Mrpl21	Spire1	Fam133b	L3hypdh
Gm10012	Lrrc40	BC003965	Chek1	Ehmt1	Dnase1l1
Espl1	Tug1	Ppfia1	Polr2a	Rtel1	Pum1
Sfswap	Hk2	Rock2	Ulk1	Zfp628	Casc5
Cdc25c	Vangl1	Akap13	Maml1	Cdc20	Pigo
Chchd6	Gid4	Cit	Ccbl2	Csnk1d	Hbs1l
Taf15	Ccdc173	Trp53cor1	Kpnb1	Phf2	Upf1
Arhgap11a	Lrfn4	Gpsm2	Tbc1d2b	Gtf2ird2	Dus1l
Rnf169	Mki67	Thrap3	Macf1		

Table S2 (Relating to Figure 6): Gene list of intersection between 3000 genes that contributed most to PC2 and 2500 most DEG between RNAseq datasets from Oxa-treated WT and Gli2∆N2 skin CD4 T cells.

S1pr1	Etf1	Pycard	Rbm6	Ercc6l	Sell	Dusp10	Trim25	Ccm2	Pik3c3	Stk38
Ptcd3	Vps37b	Bysl	Rbbp4	Itgb1	Nfkbie	Dffb	Ncaph2	Pik3cd	Gramd3	Traf5
Slamf6	Ccr7	Zbtb6	Atp1b3	Mgst2	Tmem71	Btg2	Slfn9	Canx	Nsg2	Smc3
Trib2	Vps36	Nfkbiz	Chek2	Add3	Fam65b	Fam134b	Sap30l	Chordc1	Bcl10	F2rl1
Stt3b	Itga4	Mta2	Alyref	Cdk7	Nrp1	Map4k4	Trmt10a	Susd6	Utp18	Abce1
Klhl6	Gm17296	Rexo4	Rnf19a	Crlf3	Anp32a	Cggbp1	Rpf2	Myo1f	Nhp2l1	Cse1l
Vcp	Parvg	Xrn2	Ptpra	Eif3m	Fam49a	Zfp605	Sf3b3	Mfsd6	Slc25a33	Clint1
Ldlrap1	Ubr7	Zfp874a	Sidt1	Ccdc25	Ppa1	Frmd4b	Xrcc5	Snrpg	Epb4.1	Asap1
Gphn	Hnrnpa2b1	Rasgrp2	Tcf7	Ttc13	Uba1	Pde2a	Nomo1	Tgif1	Tfe3	Keap1
Prkcq	Eif2b3	Rbm44	Tuba4a	Eif4a1	Stip1	Elf1	Tox4	Slain2	Zc3hav1	Pwp2
Gpd1l	Tcp1	Cdc42se1	Ndufb2	Carhsp1	Aldh18a1	Cradd	Trp53	Hsph1	Kdm5b	Bcl2a1b
Adss	Zfp748	Noc2l	Isca1	Galnt12	AB124611	Dtx3l	D930028M14Rik	Catsperd	Rras2	Tktl1
Mrps5	Ncoa4	Tnks2	Gltp	Cenpo	Hmgb2	Sp110	Phf5a	Ddx31	Me2	Plrg1
Usp38	Gtpbp4	Uso1	Mgat2	Idh3a	Stat5b	Aggf1	Ss18	Sipa1	Api5	Lrrn4
Psmc2	Gtf2i	Kcnab2	Mto1	Dis3	Spidr	Atp5b	Csde1	St6galnac1	Cstf3	Hspa9
Zfr2	Zfp12	Gnpat	Il4ra	Nup37	Sh3bgrl	Rnf4	Ehhadh	Pik3r5	Rnmt	Fbxo17
Rcbtb1	Dusp5	Dpy19l1	Ticrr	Lcp1	Rrp9	Serpinc1	Cpt1a	Psma6	Arl6ip1	Gramd1b
Lipa	Med18	Tesk1	S1pr4	Rangap1	Slc4a7	Wsb1	Tnfaip3	Rnf187	Dennd2d	Tnfsf14
Pxylp1	Hspa4	Prdm16	Rassf2	Ndufaf1	Frg1	Ubfd1	Entpd5	Polr2b	Eif4h	Cytip
Nr4a2	Cmah	Suco	Mcm6	Mrpl12	Madd	Fam175b	Plscr1	Fasn	Lrrc41	Ppa2
Nasp	Stk4	Akt2	Smarca5	Ifnar1	Zc3h12a	Jak1	Inafm2	Atad2b	Gm10336	Uqcrc2
Lfng	Gpr132	Hprt	Ripk2	Mthfd1l	Nefh	Ccng1	Eif3l	Acsl5	Marveld2	Blmh
Chdh	Kars	Nadk2	Gramd1c	Gimap3	Papolg	Trpc4ap	Cct2	Msn	Galnt10	Cenpa
Slfn2	Cnot1	Phrf1	Tnfrsf26	Sla	Crebrf	Tomm70a	Llph	Fen1	Arhgap1	Nap1l4
Fbl	Ets1	Ap1b1	Srfbp1	Cd226	Rbmxl1	Evi2a	Rab3gap1	Rasa3	Nol12	Eif2s1
Fcf1	Ndufa4	Wdr3	Zbtb11	Il2	Xpnpep1	Tubgcp4	Rpl21	Satb1	Top2b	Atp11b
Exoc6	Hspa14	Rnpep	Zfp472	Slc44a2	Cdc5l	Snx1	Slc9a9	Rbm10	Nars2	U2af1
Orai1	Twistnb	Gm1966	Lrrc32	Aqr	Xcr1	Nup107	Actr3	Msh6	Rpa1	Rom1
Nop2	Lsm6	Supv3l1	Dnmt1	Pole	Cdc25b	Atp2a2	Gars	Atg4d	Arl5c	Ndufb5
Tapt1	Agfg1	Cenpm	Arhgap15	Stat4	Wdr12	Pus7l	Fbxo22	Ppp1cc	Zfp101	Psat1
Polg2	Skiv2l2	Cyfip2	Tdrp	Mri1	Unc79	Polr2j	Sar1a	Rpp40	Zfp119a	Gimap4
Fh1	Fhit	Galnt7	Siglecf	Spryd4	Ppp2r5c	Slc19a1	Rnf125	Msl3	Psmd14	Cep78
Pygb	Ppif	Mocs3	Lpar3	Bcl2a1d	Smap2	Zkscan14	Crem	Agap3	Traf4	Tpm4
Nnt	Tfip11	Zbtb24	Cryl1	Spn	G3bp1	Mis12	Ldha	Rps15a	Gpbp1	Secisbp2
Gtf3c1	Prkra	Vipr1	Acpp	Rars	Ndufs4	Sh3kbp1	Chchd1	A930005H10Rik	Ift80	Ramp3
Uspl1	Cmas	Slc12a7	Rtn1	Lpin2	Gabpb1	Nup210	Casc1	Tbc1d1	Dhfr	Dpp4
Stk17b	Ppp2ca	Iba57	Herc4	Ankrd13a	Pcid2	Mettl4	Ece2	Npas1	Myd88	Ndc80

Trp53inp1	Spata2l	Smg8	Sema3e	Nxnl1	Rsl24d1	Tpp2	Sub1	Rab8a	Acss1	Dscc1
Lbr	Cog6	Zap70	Ier5	Zscan29	Prmt5	Txnrd1	Mpzl3	Ccdc53	Mfsd2a	B3gat3
Wdr75	Thg1l	Ermn	Ifngr1	Simc1	Nolc1	Nab2	Fgd6	Rbm19	Pbdc1	Gcat
Snhg6	Il2ra	B3galt4	Dpp8	Zfp456	Cabin1	Tbrg4	Sart1	Tmem64	Capn11	Prkacb
Ppat	Fam129a	Snip1	Actg2	Pnpla2	Gtf2b	Mrpl1	Eif3d	Farsb	Glipr2	Nup153
Maff	Helz	Mboat7	Pramef8	Eif3g	Dus4l	Tmem186	Lrrc20	Fam78a	Samhd1	Agpat1
Cd1d1	Fastkd1	Grb7	Arl5b	Tbc1d9b	Gpr18	Zswim3	Nbn	Rpl7l1	Stag1	Pycr2
BC030867	Gadd45gip1	Rpf1	Ruvbl2	Srprb	Mov10	Slc16a3	Psmb10	Hspd1	Top2a	St6galnac4
Prep	Nfatc1	Colq	Hmgn2	Hsp90b1	Ppan	Galk2	Srsf7	Chtop	Atg4b	Snord33
Cep72	Nsl1	Acss2	Rsl1	Dennd4c	Sp140	Lpxn	Zrsr1	Hnrnpc	Capza1	Brix1
E2f2	Cndp2	Gm14634	Ssrp1	Gemin5	Cnbp	Kpna3	A630072M18Rik	Serpinb9	Ghitm	Carf
St8sia6	Lrrc66	Gen1	Rnf225	Bub1	Ppp2r1b	Vta1	Coq10b	Zmat1	Rpl37a	Pes1
Seh1l	Slc35e1	Zswim4	Zbtb45	Dtx1	Usp14	Lsm11	Cox17	Gabrr2	Rragd	Sestd1
Flna	Dnph1	Ndrg1	Kif11	Prmt3	Smarcd2	Rbbp8	Ccnb1	Mlec	Rpp14	Armc3
Smtn	Spdl1	Pja1	Slbp	Ndufc1	N6amt1	Tm2d3	Asb5	Ccne2	Rassf3	Snord35b
Ccnf	Mogs	Stt3a	Wdr43	Slfn1	Chaf1b	Il17ra	Orc1	Hmces	Saysd1	Dcaf7
Tbc1d14	Larp7	Mir425	Prr5l	Cd7	Ubqln1	Ing5	Heatr5a	Fiz1	Zfp62	Mrpl18
Ppil1	Mesdc1	Pgk1	Gatad2b	Fam101b	Mrpl24	Map3k11	Sema4d	Arpc5	Mmachc	Rnf32
Abr	Ints7	Dkc1	Lysmd3	Ripk3	Arap1	Noa1	Prdm15	Tmem173	Syncrip	Rsl1d1
Iqgap2	Pgpep1l	Psmd13	Sfxn1	Ppargc1b	Actl6a	Ncl	Gch1	Med16	Slc37a1	Ankrd13b
Rnf103	Map3k5	F830016B08Rik	Xpot	Zfp574	Aunip	Rrm2	Ash2l	Atg16l2	Cactin	Igtp
Pepd	Mcm7	Noxa1	Psmb8	Lnx2	Tmem171	Abcf1	Dennd6a	Cdc42	Lyar	Myg1
Spata7	Golm1	Pcbp1	Avl9	Ern1	Hccs	Atp1a1	Rad54l	Polr1b	Gpr25	Spef1
Suv39h2	Gbp2	Ssr2	Txndc9	Ms4a6b	Fkbp15	Dnajc28	Gemin4	Hnrnph2	Il5	Tars
Terc	Cpsf3	Gtpbp1	Tnfrsf13b	Eif2s3x	Ddx46	Zfp939	Sec24d	Ms4a4b	Fbxo28	P2ry12
F8a	Champ1	U2af2	Apobec2	Mon1b	Gpr34	Acacb	Upf2	Kdm5c	Cited2	Cab39l
Jagn1	Hadhb	Lmnb1	Dusp12	Shq1	Bub1b	Aco1	Elf2	Ccdc117	Lgals3bp	Cryba4
Usf2	Fgd3	Rasd2	F630028O10Rik	Stk25	Gpx1	Apln	Il17a	Sat1	Mafb	Anxa11
Fnbp4	Lgals1	Zfp287	Ilkap	Spg7	Klhl28	Fer	AW549877	Slc4a2	Rfx2	Slc35d2
Zfp579	Srrm2	Dip2b	Scoc	Spon1	Dhx58	Rab12	Tpgs2	Lrrfip1	Alox5ap	Col5a3
Atp2b4	Hexdc	Wwc1	Smpd1	Fam102b	Tef	Fam193a	Setd6	Zfp948	Ldb1	Maf
Itm2c	Slc22a5	Csf2rb	Coq4	Atg3	Snx30	Grk4	Zfp189	Dnajc4	Sik1	Fam199x
Dbp	Kctd12	Zfp414	Cdc42ep3	Nthl1	Acadm	Scai	Tbce	Kdm4d	Rbak	Eaf2
Arl14ep	Vps13d	Araf	Areg	Syt11	Mllt6	Jund	Gtpbp2	Themis2	Per1	Slc25a51
Isca2	Ubxn8	Brd4	Fam213b	Rnu11	Mboat4	Gnai2	Atp9b	Igflr1	Tmem109	Plcb3
Rhoc	Dmrta1	Myo1c	Guk1	Dctn2	Kmt2d	Dctn5	Klf5	Rnf130	Aamdc	Ctsf

Zfp362	Rtn4ip1	Wbp5	Ppp1r12c	Aktip	Mbnl2	Atp6v0b	Efna3	Gpr62	Cd44	Exoc1
Hist1h3a	Usp11	Mapk8	Gpr160	Rasl11b	Rhbdd3	Crtc2	Ndrg2	Use1	Alg5	Snord15b
Mapkapk5	Stk19	Dnmbp	Habp4	Pqlc1	Fam117a	Itih5	Oxr1	Gm5796	Fancl	Tbc1d23
Gnb4	R3hdm2	Plcg2	Zfp511	Sh3rf1	Arhgap5	Klhl5	Peg13	Fth1	Ctla2a	Fez1
Hist1h2bp	Aldh6a1	Neb	Calca	Ctnnbip1	Amigo1	Clec9a	Rybp	Arpc5l	Fhod1	Zdhhc23
Il1r2	Ptms	Pnrc2	Gm16894	Gigyf1	Synpo	Ddx26b	Lima1	Ech1	Ryr1	Cish
Dcaf15	Rab9	Irak3	Xkr8	Ing4	Camta1	Aldh2	Ppcdc	Dennd1c	Tmem222	Cpne2
Tle6	Rab11a	Son	Atxn2l	Tmem203	Tcf7l1	Ptges3l	Tmem231	Dnajc25	Ptbp2	Gna11
Cetn4	Tpsab1	Bptf	Enpp4	Mafg	Rasgrf2	Rabac1	Ttc14	Gm20257	Rnf149	Spin1
C1rl	Gfra4	Particl	Jade1	Actr1b	Phc1	Pgrmc1	Impact	Kif3a	B930018H19Rik	Sgcb
Vps16	Rpp21	Klf7	Med15	Lat2	Nucb2	Oxld1	Cobll1	Nus1	Txndc17	Ubash3b
Hnrnpdl	Arnt2	Fabp12	Vat1	Edaradd	Slc2a1	Itga3	Ripk4	Mettl20	Zfp273	Tgfb1
Dusp6	Izumo4	Lpcat2	Fam71e1	Cdkn1b	Ccdc142	Adra2a	Egfr	Dnal4	Gemin2	Olfr56
Laptm4b	Snora41	Gad1	Chchd3	Lekr1	Kdf1	Birc2	Klf4	Lmntd2	Sult5a1	Tmbim1
Ddx3y	Ppp3ca	Mdm4	Nav2	Tmx1	Gm166	Dennd6b	Stxbp1	Fcho2	Bmyc	Tmod4
Gpx4	Tada2b	Zfp607	Zglp1	Mmp8	Tnip2	Trp53i11	Rev1	Pla2g12a	Picalm	Pip5k1c
Ppp1r1b	Slc16a4	Cldnd2	Tmem256	Dfna5	Unkl	B230219D22Rik	Snord42a	Pcdhgb6	LOC106740	Tmem216
Snora81	Chadl	Eri3	Dcbld1	Qser1	Anxa1	Fam124a	Uba5	Fst	B3gat2	Gramd1a
Ikbip	Gm15787	Mprip	Tmc3	Axl	Cbx7	Loh12cr1	Ccr8	Tnfrsf1a	Ano1	Snapin
Ptgs2	Efemp2	C330013E15Rik	Kcnk7	Cd200r1	Aqp5	Acadsb	Kif1b	Wfdc21	Tia1	Styx
Gdf5	Frzb	Ogg1	Tmed4	Il17d	Rtn4	Reg3g	Slc8a1	Tnk2	Fundc2	Snord73a
Cnot10	Hist1h1d	Ikzf2	Pid1	Cacna1g	Dclre1a	Dpep1	Phf20	Ptch1	Cd14	Meis1
Adgrl1	AI429214	Cracr2b	Tmem138	Ccnl1	Hacd1	Kifc5b	Gpc6	Duoxa1	Malat1	Snhg5
Eif4e3	Ssbp4	Eif2s3y	Zfp219	Zfp354c	Tspan11	Tmem9	Pik3r1	Ddit3	Arl4c	Chst4
Cd160	Apoe	Scamp3	Emilin2	Adarb1	Ppil6	Arrdc1	Snx7	Hist2h2ac	Nfic	Chst7
Efnb1	Slc50a1	Dusp3	Pdgfa	Igfbp4	Gm5547	Pcyox1	Pcyt1a	Vasn	Itgb8	Rasip1
Tep1	Pbx4	Ppp1r9b	Fam13c	Ift74	Rhbdf1	Pde1b	Anxa4	P3h1	Fam46c	Tmcc3
Cpsf7	Slc30a4	Ccdc157	B4galt4	Fxyd3	Cdh24	Trim29	Klrg2	Il10rb	Klrg1	Gab1
Olfm1	Tmeff2	Tceal8	Ubxn11	Fabp7	Ccl6	Scube1	Hyi	Get4	Irs2	Emp3
Nqo1	Pim1	Bcr	Atraid	Kifap3	Zfp120	Susd2	Ankrd13d	Mgst1	Smoc2	Ppp1r3c
Ankrd37	Cfap36	Gm5088	Cadm1	Plcl1	Rnf217	Tspan2	Gfpt2	Tubg2	Pts	Ace
Maob	Rai14	Tcf15	D830031N03Rik	Rab37	Col1a2	Klhl4	Prss35	Wls	Irx2	Mir5107
Sncg	Zkscan3	Diap1	Erdr1	Fkbp3	Fgd5	Bnc2	Vegfb	Ecm1	Apobec3	Tox
Lgals6	Whamm	Pik3r3	Kcne4	Acot12	Plekhm2	Arg2	Galc	Fam180a	Hecw2	Plcd1
Zfp382	Rc3h1	Fermt1	Ppp2r5b	Robo4	Nisch	Fkbp1b	Evi5	Pttg1ip	Slc5a3	Irx5
Man2c1os	Dstn	Btbd19	Tubb6	Rasal2	Glis2	Smim20	Chmp6	Slc6a4	Gm16712	Sox6
Timp2	Thbs1	Gm10416	Egr1	Tmem54	Kcna3	Rhob	Cnn3	Arhgef15	Sash1	Adh7

Fgfrl1	Itfg3	Col1a1	Olfml2b	Trip10	Lgr4	Pdzk1	Ube2g1	Gadd45g	Ttc16	Id2
Dock9	Ift81	Cebpzos	Fgl2	Sgce	Hdc	Sectm1a	Rnf11	Lincpint	Ttc30b	Mxra8
Mpped2	Zak	Spag17	Atrx	Gjb4	Dynlt1b	Ptprb	Ptpn23	Sparcl1	Xlr4c	Dynlt3
Tmem60	Cd109	Ddit4	Rasgrp3	Cd8a	Rcan1	Zc3h7a	Fut7	Ankrd16	Scn11a	Egln3
Scamp1	Rgcc	Siah2	Ntrk2	Sdr16c6	Eid1	Rnf170	Kmt2c	Nts	Sgms1	Ptpn5
Slc35e4	Tspan15	Fxyd7	Zfhx4	Mfsd9	Azin2	Adgrg1	Rab6a	Sphk1	Fut8	Inha
Sepn1	R3hdm1	E230029C05Rik	Gsn	Cmtm7	P3h3	Edn3	Gap43	Ltbp3	Fbxl12	Atp1a2
Mapre3	Phlda1	Pgs1	Cep164	Slc25a38	Baiap3	Adamts4	Stx4a	Adgra2	Adam15	Cd200r4
Lrrn2	Nicn1	Efnb2	Clip2	Fgf7	Mdk	Sox5	Mir7079	Trmt13	Timp4	Cxcl5
Rac3	Magi1	Htra4	Kif27	Fam132a	Palld	Sod2	Creb3l4	S100a6	Galt	Id4
Zdhhc1	Pdzd2	Bcar1	Fam213a	E230013L22Rik	Pcp4l1	Eif4a2	Mia	Arhgef28	Col4a1	Gem
Arhgap21	Gm2a	Tacstd2	Atpif1	Tspan18	Il10	Acaca	Rgs5	Gja4	Arl5a	Gstm7
Plagl1	Arl4a	Pdp1	Ptov1	Gm9776	Dcakd	Ndfip2	Zc2hc1a	Ciart	Trove2	Adam8
Gstt1	Col16a1	Apc	Phactr2	Klf9	Il9r	Prickle3	C1ra	Praf2	Megf6	Slc35f5
Lpar1	Clec2d	Fbln2	Ldlrad3	Gm20605	Gpihbp1	Rell1	Pi16	Dusp22	Lhfp	Igf1
Cald1	Islr	Msx1	Gli2	Pcolce	Pcbp4	Adgrg3	Scd1	Ulk2	Mmp23	Me1
Clk2	Ltbp2	Fkbp7	F5	Ccdc102a	Adamts2	Thbs2	Gstm4	Ebf1	Entpd2	Jun
Tmed10	Fgfr1	Btg1	Loxl3	Pdgfrb	Cyb5r3	Foxe1	Hoxaas2	Dlx5	Klhl29	Ly6g6c
Dusp1	Ptgis	Lims2	Slc27a3	Csf1	Leprot	Mast4	Ebi3	Fzd9	Lmo1	Gpr4
Ccdc3	Peg12	Acap1	Dgat2	Trim16	Tnnt1	Ap3m2	Cyp27a1	Ddr2	Mrc2	Ccnl2
Ncmap	Lpin3	Ormdl3	Mmrn2	Clec11a	Epc1	Polm	Tagln	Ghr	Fam19a5	Gm14005
Msrb3	Parp4	Gstm1	A430105I19Rik	Tex9	Lman2l	E4f1	Sorbs3	Eva1b	Dact3	Etl4
Crip2	Dtx3	Ift43	Col3a1	Zmym6	Col2a1	Pou3f1	Pxdc1	Phf21a	Fbxo25	Spats2
Hes1	E230016M11Rik	Zfp36	Kdr	Ptn	Gm17745	Jkamp	Capns2	Ucma	Tmem134	Pvrl3
Pcgf2	Trf	Syt15	Crmp1	Hrh1	Ralgapa1	Npr1	Siah1a	Pcdhb9	Unc119	Pdlim7
Krt5	Uggt2	Ehd2	Lox	Scara5	Dlgap4	Cxcl2	Ptprv	Cd302	Pmp22	Bmp6
Rab24	Ophn1	Dpysl3	Lyz1	Chad	I730030J21Rik	Itgb5	St3gal2	Apold1	Ndn	Sgms2
Krt28	Sdc4	Hist1h2ac	Dennd5b	Caskin2	Cx3cl1	Emp2	Rhbdl2	S100b	Camk1	Gcnt2
Sult1a1	Sprr2a3	Il13ra1	Fos	Slc39a13	Klf6	Stap2	Naprt	Slc16a9	Ceacam1	Sectm1b
Afap1l1	Uty	Srsf5	Vstm4	Msantd3	Des	Fcgrt	Snx9	Mylk	Hist1h2bg	Myoc
Dmd	G0s2	Pde4c	Gata2	Grb10	Sox17	Bmpr1a	Comp	Cyp1b1	Cemip	Pkig
Zfp28	Ecm2	Sfrp2	Slfn8	Vim	Car7	Hist1h1c	Prkar1b	Tbcb	Slc26a8	Slc20a2
Nfia	Mall	Trp63	Fmo1	Twist2	Rgs14	Col20a1	Arhgap29	Ogn	Gfap	Pag1
Tmem255b	Tjp1	Cd81	Cdh13	H1f0	Fmod	Osr2	Armcx1	Wdr34	Bmi1	Ppap2b
Dpysl2	Mecom	Pcsk1	Crispld2	Mtss1l	Prr29	Epn3	Acot1	Ido1	Car8	Egfl7
Lzts2	Cox4i2	Stx2	Bmp5	Aldoart1	Lyz2	Ushbp1	Col6a3	Apod	Sox9	Aldh3a1
Notch3	Olfr558	Tmtc3	Fam171a1	Ypel3	Zbtb10	Cfh	Fam217b	Cdo1	Pcdh12	Fkbp14

Pth1r	Ptk7	Lrrc17	Npr2	Ccdc85b	Tmem44	Cgnl1	Nfib	Dsc3	Ago1	Ccnd1
Thbs4	Cda	Rgma	BC031361	Rian	Sap25	Kank3	Gp1bb	Pvrl2	Gm19466	Wif1
Chil1	Serpinf1	D6Ertd527e	Tle2	Spns2	Sh3bgrl2	Adcy9	Gpha2	Fzd1	Paqr6	Twist1
Lamc3	Col12a1	Pdgfrl	Rab40b	Arhgef19	Dkk3	Peg3	Pla2g5	Antxr1	Mmp16	Wtip
Meis2	Nexn	Mfap3l	Klra2	Eln	Hpgd	Corin	Col6a2	Luc7l2	Hilpda	Plp1
Mpz	Nrn1	Gm10406	Tpm2	Lmna	Rgs11	Prrt2	F11r			

Table S3 (Relating to Figure 6): Expression values of Shh and Gli3 in RNAseq datasets from Oxa-treated WT, Gli2ΔC2 and Gli2ΔN2 skin CD4 T cells.

	1 WT-CD4	2 WT-CD4	1 Gli2ΔC2-CD4	2 Gli2ΔC2-CD4	1 Gli2ΔN2-CD4	2 Gli2ΔN2-CD4
Shh	0	0	0	0	0	0
Gli3	0	0	0	0	0	0

Table S4: (Relating to Materials and Methods): Table shows mouse and human antibodies, their clones and the company where they were purchased from.

	Antibody	Clone	Company
Mouse	antiSmad2+Smad3 (Phospho T8)	Ab63399	Abcam
	CD3	17A2	Biolegend
	CD4	RM4-5	Biolegend
	CD8	53-6.7	Biolegend
	CD11b	M1/70	Biolegend
	CD11c	N418	Biolegend
	CD44	IM7	eBioscience
	CD45	30-F11	Biolegend
	CD62L	MEL-14	eBioscience
	CD69	H1-2F3	Biolegend
	CTLA-4	UC10-4B9	Biolegend
	donkey anti-rabbit PE secondary antibody	Poly4064	Biolegend
	F4/80	BM8	Biolegend
	Foxp3	MF-14	Biolegend
	IFN-γ	XMG1.2	Biolegend
	IL4	11B11	Biolegend
	IL5	TRFK5	Biolegend
	IL10	JES5-16E3	Biolegend
	IL13	eBio13A	eBioscience
	IL17	TC11-18H10.1	Biolegend
	Ki67	16A8	Biolegend
	Klrg1	2FI/KLRG1	Biolegend
	LAP	BC96	Biolegend
	MHCII	M5/114.15.2	Biolegend
	SiglecF	E50-2440	BD Pharmigen
	γδTCR	GL3	Biolegend
Human	CD4	RPA-T4	Biolegend
	CD25	BC96	Biolegend
	FOXP3	236A/E7	Biolegend
